# Multimeric Scaffolds Displaying the HIV-1 Envelope MPER Induce MPER-Specific Antibodies and Cross-Neutralizing Antibodies when Co-Immunized with gp160 DNA

**DOI:** 10.1371/journal.pone.0113463

**Published:** 2014-12-16

**Authors:** Shelly J. Krebs, Sean P. McBurney, Dina N. Kovarik, Chelsea D. Waddell, J. Pablo Jaworski, William F. Sutton, Michelle M. Gomes, Maria Trovato, Garret Waagmeester, Susan J. Barnett, Piergiuseppe DeBerardinis, Nancy L. Haigwood

**Affiliations:** 1 Division of Pathobiology and Immunology, Oregon National Primate Research Center, Oregon Health and Sciences University, Beaverton, OR, United States of America; 2 Institute of Protein Biochemistry, C.N.R., Naples, Italy; 3 Viral Vaccines Program, Seattle Biomedical Research Institute, Seattle, WA, United States of America; 4 Novartis Vaccines & Diagnostics, Emeryville, CA, United States of America; Simon Fraser University, Canada

## Abstract

Developing a vaccine that overcomes the diversity of HIV-1 is likely to require a strategy that directs antibody (Ab) responses toward conserved regions of the viral Envelope (Env). However, the generation of neutralizing Abs (NAbs) targeting these regions through vaccination has proven to be difficult. One conserved region of particular interest is the membrane proximal external region (MPER) of Env located within the gp41 ectodomain. In order to direct the immune response to this region, the MPER and gp41 ectodomain were expressed separately as N-terminal fusions to the E2 protein of *Geobacillus stearothermophilus*. The E2 protein acts as a scaffold by self-assembling into 60-mer particles, displaying up to 60 copies of the fused target on the surface. Rabbits were immunized with E2 particles displaying MPER and/or the gp41 ectodomain in conjunction with DNA encoding full-length gp160. Only vaccines including E2 particles displaying MPER elicited MPER-specific Ab responses. NAbs were elicited after two immunizations that largely targeted the V3 loop. To overcome V3 immunodominance in the DNA component, E2 particles displaying MPER were used in conjunction with gp160 DNA lacking hypervariable regions V2, V3, or combined V1V2V3. All rabbits had HIV binding Ab responses and NAbs following the second vaccination. Using HIV-2/HIV-1 MPER chimeric viruses as targets, NAbs were detected in 12/16 rabbits after three immunizations. Low levels of NAbs specific for Tier 1 and 2 viruses were observed in all groups. This study provides evidence that co-immunizing E2 particles displaying MPER and gp160 DNA can focus Ab responses toward conserved regions of Env.

## Introduction

HIV-1 infection continues to be a worldwide epidemic with more than 2.5 million individuals newly infected annually [1]. In order to stop the spread of HIV-1 infection and ultimately control the pandemic, a vaccine is considered a critical tool. A major challenge of HIV-1 vaccine development has been the large diversity of viral isolates. Numerous strategies have been developed to address viral diversity [for review [2]–[4]], and one strategy of particular interest is to focus on highly conserved regions of Envelope (Env) that are targets of neutralizing (N) antibodies (Abs) during infection. Over the last two decades a number of human neutralizing monoclonal Abs (NmAbs) that map to conserved regions of Env have been isolated from HIV-positive subjects with potent NAbs. Such NmAbs have been shown to neutralize >90% of viral isolates tested [5], and are thus termed broadly neutralizing Abs (bNmAbs). These bNmAbs are capable of protecting against infection in challenge models [6]–[10].

The membrane-proximal external region (MPER), located within the gp41ectodomain, is one of the most conserved regions on Env. It contains epitopes of three well-characterized human bNmAbs: 2F5, 4E10, and 10E8 [11]–[16]. Of these, 4E10 and 2F5 are the most fully characterized for their functionality. The MPER plays a role in membrane fusion, and both 2F5 and 4E10 block a crucial step in the fusion-intermediate state between the viral and target cell membranes [11], [15], [17]. Besides V3, most of the gp120 epitopes recognized by characterized mAbs are conformational, nonlinear epitopes, whereas bNmAbs 2F5 and 4E10 recognize contiguous epitopes in a linear sequence within MPER [15], making it an attractive target to exploit for vaccine design. Studies of cross-clade neutralization have shown that 2F5 and 4E10 neutralize diverse primary isolates at 67% and 100%, respectively [18], and viruses cloned directly from newly infected patients at 80% and 100%, respectively [19]. The recently discovered bNmAb 10E8 has similar potency and breadth, neutralizing 98% of viruses tested [16]. Passive immunization with 2F5 and 4E10 conferred sterilizing protection against infection in animal models [9], [20], providing evidence that the presence of these Abs in the appropriate concentrations prior to infection may protect against transmission of primary isolates [21]. A previous concern about the use of this region as a vaccine was the evidence that both 2F5 and 4E10 NmAbs recognized autoantigens. This concern has been greatly diminished by the discovery of 10E8, which does not demonstrate autoreactivity [16].

Although non-NAbs may be associated with vaccine efficacy, as was seen in the most recent Phase III RV144 Thai trial demonstrating transient protection [22], [23], it is anticipated that vaccines that elicit bNAbs would be of great value because they are likely to increase the potency and breadth of vaccine protection. Therefore generation of bNAbs has been the major focus of immunogen design. Vaccines designed to target conserved gp41 epitopes have been tested in many previous studies [24]–[29], [30], and while most were able to elicit Ab responses, only a few immunogens have generated low level NAbs in small animals [31]–[33], [34]–[36] or protection *in vivo* in macaques via trancytosis-blocking Abs [37].

Previous studies in our lab have explored the use of a multimeric scaffold based upon a subunit of the pyruvate dehydrogenase (PDH) complex of *Geobacillus stearothermophilus* to display regions of HIV-1 Gag and Env [38], [39]. Sixty copies of E2 self-assemble into a pentagonal dodecahedral scaffold with icosahedral symmetry, resulting in the formation of a large multimeric particle with a molecular weight >1.5 MD and a diameter of approximately 24 nm [40], [41]. Due to the heat-stable properties often found in proteins from thermophilic bacteria, the E2 protein can be renatured *in vitro* from denaturing conditions to form the 60-mer scaffold without the need of chaperonins [42]. This protein scaffold can be modified on the N-terminus by replacing the natural peripheral domains of E2 with foreign peptides and proteins, creating a novel E2 multimeric antigen display system. Moreover, the E2 particle naturally associates with 60 copies of the E1 (150 kDa) or E3 (100 kDa) enzymes non-covalently on its surface [43], thus up to 60 polypeptides can be presented on the E2 scaffold as N-terminal fusion proteins without negatively impacting the native folding of the E2 core. Conceptually, multimerization may improve immunogenicity by providing bivalent binding opportunities for Abs. Also, the E2 particles are devoid of any viral genetic material or viral enzymes and thus have the potential to be safer than attenuated or inactivated wild-type viruses when used as immunogens in humans.

Using this system, we showed previously that the E2 multimeric scaffold displaying Gag p17 elicits Gag-specific Abs and T cell responses in mice [38]. When used to display the third hypervariable loop (V3) of HIV-1 Env, E2 particles induced V3-specific NAbs in rabbits and T cell responses in mice [39]. In that study, simultaneous co-immunization of these particles with gp160 plasmid DNA was more effective than each individual component alone or the DNA prime, protein boost immunization regimen, even in the absence of adjuvant [39]. In this current study, we designed E2 particles displaying the gp41 ectodomain or MPER as N-terminal fusion proteins, with the goal of eliciting NAbs that were broadly neutralizing and directed to MPER.

E2 particles displaying MPER or gp41, or both, in combination with gp160 plasmid DNA, were immunogenic and elicited equivalent gp41-specific Abs; however, only rabbits immunized with E2 particles displaying MPER developed significant binding Abs directed to MPER. All immunized rabbits developed NAbs to HIV-1 SF162 after two immunizations, but the majority of these NAbs were directed to the immunodominant V3 loop. In order to overcome the immunodominance of V3 and other hypervariable regions, gp160 constructs with deletions in the V2, V3, or combined V1, V2, and V3 regions were used in conjunction with the MPER-E2 fusion particles and compared to immunization with full-length (wt) gp160 DNA. The resulting vaccines elicited similar levels of gp140 binding Abs. Epitope mapping of these Abs revealed that the deletion of V3 resulted in increased recognition of MPER peptides compared to the full-length gp160, and increased titers of NAbs after four immunizations. All other groups had equivalent levels of NAbs to HIV-1 SF162. Additionally, 12/16 rabbits immunized with DNA plus MPER-E2 particles developed Abs capable of neutralizing HIV-2/HIV-1 MPER chimeric viruses, but this activity was not correlated with binding to MPER peptides. Sera from all groups demonstrated low-level neutralization of selected Tier 1 and Tier 2 viruses after two or three vaccinations, and the NAbs had greater breadth than those elicited in our prior studies with E2 particles displaying V3 [39]. Collectively these results indicate that immunization with E2 particles displaying MPER in combination with gp160 Env plasmid DNA is capable of directing Ab responses to MPER and eliciting moderately cross-reactive NAbs.

## Materials and Methods

The Oregon Health & Science University West Campus Institutional Animal Care and Use Committee approved this research under protocol number IS000954.

### Rabbit Immunizations

Female New Zealand White rabbits (Western Oregon Rabbit Company, Philomath, OR) were housed at the Oregon National Primate Research Center (ONPRC) at Oregon Health & Science University in accordance with the standards outlined by the National Institutes of Health Guide for the Care and Use of Laboratory Animals. All studies were performed according to the rules approved by the Institutional Animal Care and Use Committee (IACUC). The ONPRC is an AAALAC-accredited institution. The first study described here was completed with nine rabbits, with three rabbits per group. Two additional rabbits were immunized three times with E2wt particles to generate E2-specific serum. The effect of modifications to the gp160 vector was studied subsequently using 16 rabbits with four rabbits per group. Rabbits were immunized intramuscularly with a total of 200 µg of protein per immunization with Incomplete Freund's adjuvant (IFA). Codon-optimized SF162 gp160 DNA, either full-length or lacking variable loop regions 2, 3, or combined V1, V2, and V3, was delivered intradermally via Gene Gun (Bio-Rad) at a pressure of 400 psi. A total of 36 µg of DNA was delivered in 18 shots of 2 µg DNA each, given in clusters of three non-overlapping positions at six shaven sites (lower back, inside of back legs, and abdomen). Blood was collected and serum was separated and heat inactivated at 56°C for 1 h before being stored at −20°C.

### Construction of HIV-1 Env-E2DISP plasmids

**Figure 1:**
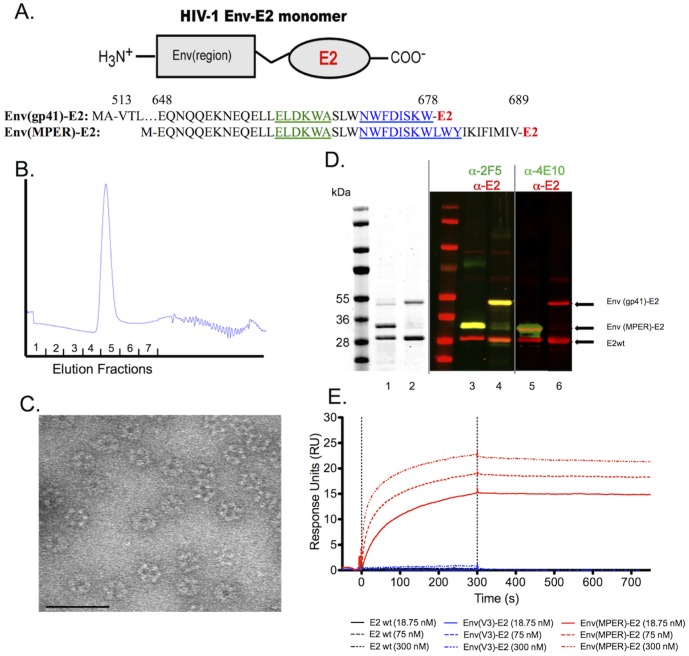
Construction and antigenic characterization of the HIV-1 Env(MPER)-E2 and Env(gp41)-E2 particles. (A) Schematic illustration of an E2 monomer with the HIV-1 Env region fused to the N-terminus. Amino acid sequences of the Env(MPER)-E2 and Env(gp41)-E2 constructs are shown below and numerated in relationship to the reference strain HXB2. Within the amino acid sequences, the 2F5 mAb epitope is underlined and highlighted in green and the 4E10 mAb epitope is underlined and highlighted in blue. (B) Representative SuperdexX200 gel filtration chromatograph of purified Env(MPER)-E2 particles. (C) Representative transmission electron micrograph of HIV-1 Env(gp41)-E2 particles. Env(MPER)-E2 and E2wt particles were of similar size and shape. Scale bar is 75 nm. (D) SimplyBlue stain of Env(MPER)-E2 (lane 1) and Env(gp41)-E2 (lane 2) revealing the purity of these particles at 93% and 90%, respectively. Western blot analysis of Env(MPER)-E2 (lanes 3 and 5) and Env(gp41)-E2 (lanes 4 and 6) using the Li-COR Odyssey dual-detection system to demonstrate the identity of these particles. Red bands indicate proteins detected with anti-E2 Abs, and green bands indicate proteins detected by either 2F5 or 4E10 mAbs. Yellow bands indicate proteins that are detected by both anti-E2 and either 2F5 or 4E10 mAbs. (E) Surface Plasmon Resonance Spectroscopy of Env(MPER)-E2 particles binding to the 2F5 NmAb to demonstrate accessibility of the 2F5 epitope on the surface of the scaffold. Binding was detected by flowing 2F5 mAb concentrations of 18.75 nM, 75 nM and 300 nM over E2 particles immobilized on a CM3 chip. No binding of the E2wt scaffold (lacking HIV fusion proteins) or Env(V3)-E2 particles to the 2F5 mAb were detected.

The Env(MPER)-E2 and Env(gp41)-E2 expression vectors were constructed from the previously described pETE2DISP plasmid [40]. The oligonucleotide sequence encoding the SF162 Env MPER peptide (amino acid (aa) 648–689, HXB2 numbering) or gp41 extracellular region (aa 513–678, HXB2 numbering) was cloned into the pETE2DISP vector for expression of the Env epitope as an N-terminal fusion to the E2 core scaffold (Fig. 1A). The oligonucleotide sequence encoding MPER was amplified using primers GCGCCCATGGAACAGAACCAACAAGAAAAG and GCGCCCCGGGTACTATCATTATGAATAT, and the oligonucleotide sequence encoding the gp41 extracellular region was amplified using primers GAGAAGAGTGGTGCAGAGAGAAACCATGGCAGTGACGCTAGGAGCTATG and CATTATCCCGGGTTTTATATACCCGGGCCATTTTGATATGTCAAACC; each containing the restriction sites *NcoI* and *XmaI.* Cycling conditions for the PCR were as follows: denature at 94°C for 2 min, 10× (94°C for 15 sec, 49°C for 30 sec, and 72°C for 60 sec), 20× (94°C for 15 sec, 65°C for 30 sec, and 72°C for 60 sec), and a final elongation of 72°C for 7 min. The PCR product and the pETE2DISP vector were double digested with *NcoI* and *XmaI* (New England Biolabs) and ligated together with T4 DNA ligase (New England Biolabs) before transformation into BL21 (DE3) CodonPlus-RIPL competent cells (Stratagene). In-frame ligation of all constructs was confirmed by sequencing. These constructs encoding N-terminal fusions of MPER or the gp41 extracellular region to the E2 scaffold are annotated in this document as Env(MPER)-E2 and Env(gp41)-E2, respectively.

### Expression, purification and refolding of Env-E2 multimeric scaffolds

Plasmids encoding E2 wild-type (E2wt) (i.e., scaffold protein without an HIV-1 N-terminal fusion) and Env-E2 fusion proteins were maintained and expressed in BL21 (DE3) CodonPlus-RIPL cells (Stratagene). Cells were grown overnight at 37°C in Luria-Bertani (LB) broth with a final concentration of 100 µg/ml ampicillin and 50 µg/ml of chloramphenicol, shaking at 225 rpm. The following day, the cultures were back diluted 1∶20 and grown to an OD_600_ between 0.8–1.0. Protein expression was induced with the addition of Isopropyl β-D-1 thiogalactopyranoside (IPTG) with a final concentration of 1 mM. Cells were harvested by centrifugation at 5000 g for 5 min at 4°C. Pellets were then resuspended in Lysis buffer (1× BugBuster Protein Extraction Reagent (Novagen), 1xPBS, Benzonase Nuclease (EMD), rLysozyme (EMD), TurboDNase (Ambion), Cyanase (RiboSolutions) and Complete, EDTA-free Protease Inhibitor Cocktail Tablet (Roche)) and incubated at room temperature for 30 min and then at 37°C for 30 min, shaking at 225 rpm.

The soluble fraction containing the E2 monomers was recovered after centrifugation at 10,000 g for 10 min at 4°C and was loaded onto a Sephadex G-25 column (GE Healthcare) for buffer exchange. Fractions containing E2wt were pooled and loaded onto a Detoxi-gel column (Pierce). E2wt did not bind to the Detoxi-gel column and was recovered in the flow through, which was loaded onto a Q-Sepharose anion exchange column (GE Healthcare) at 1.0 ml/min. Bound protein was eluted from the column with a 0–60%/400 ml gradient of elution buffer (20 mM Tris-HCl, pH 8.5, 1 mM EDTA, 1 M NaCl) at 3.0 ml/min., with the E2wt eluting at a salt concentration of 30%. Peak fractions containing E2wt were pooled and concentrated with a 10 kD molecular weight cut off (MWCO) using Amicon Ultra Centrifugal Filter (Millipore). The retentate was loaded onto a Superdex200 gel filtration column (GE Healthcare) at 1 ml/min using Solubility Buffer 2.2 (1× PBS, 50 mM L-Glutamine (Sigma), 50 mM NaCl, 250 mM L-Arginine (Sigma)). Fractions containing the 1.5 MDa E2wt 60-mer particles were concentrated to 1 mg/ml using the Ultra Centrifugal devices and then stored in Solubility Buffer 2.2 at −80°C.

The Env-E2 fusion proteins formed inclusion bodies in *E.coli* and were purified from the insoluble fraction following bacterial lysis and centrifugation as described above. Inclusion bodies were washed three times with Inclusion Body Wash Buffer (1 M Guanidine Hydrochloride (GuHCl), 50 mM NaCl, 1 mM DTT, 1× PBS, 10% glycerol, 0.5 M Arginine, pH 7.4) before dissolving in Unfolding Buffer (6 M GuHCl, 1 mM DTT, 1× PBS). To produce soluble HIV-1 Env-E2 multimeric particles, E2wt was combined with the Env-E2 inclusion bodies at either a 1∶1 (Env(MPER)-E2:E2wt) or 1∶3 (Env(gp41)-E2:E2wt) molar ratio and allowed to unfold in Unfolding Buffer (6 M GuHCl, 1 mM DTT, 1× PBS) rocking at 4°C for a minimum of 3 h. The proteins were transferred to SnakeSkin Dialysis Tubing, 10K MWCO (Pierce) and subjected to step-down dialysis against the following buffers: 4 M GuHCl (4 M GuHCl, 50 mM NaCl, 1 mM DTT, 1× PBS, 10% glycerol, 0.5 M Arginine, pH 8.0), 2 M GuHCl (2 M GuHCl, 50 mM NaCl, 0.5 mM DTT, 1× PBS, 10% glycerol, 0.5 M Arginine, pH 8.0), 0 M GuHCl#1 (50 mM NaCl, 1 × PBS, 10% glycerol, 0.5 M Arginine, 0.5 mM reduced glutathione, 0.1 mM oxidized glutathione, pH 8.0), 0 M GuHCl#2 (50 mM NaCl, 1× PBS, 10% glycerol, 0.5 M Arginine, 0.1 mM reduced glutathione, 0.1 mM oxidized glutathione, pH 8.0), and 0 M GuHCl#3 (50 mM NaCl, 1× PBS, 10% glycerol, 0.5 M Arginine, pH 8.0). A final dialysis was performed in Solubility Buffer 2.2. Refolded soluble 60mer particles were confirmed by gel filtration using the Superdex200 gel filtration column (GE Healthcare), and purity and identity were assessed by SDS-PAGE and Western blot analysis, respectively. Purified multimeric proteins were stored at −80°C.

### SDS-PAGE and Western blot analysis

Expression, refolding, and identity of recombinant proteins were assessed by SDS-PAGE and Western blot analysis using Invitrogen NuPAGE 4–12% Bis-Tris mini-gels (Carlsbad, CA) under reducing conditions. For SDS-PAGE, gels were stained with SimplyBlue SafeStain (Invitrogen). For Western blot analysis, proteins were transferred onto nitrocellulose paper (Invitrogen), blocked with Odyssey blocking buffer (LI-COR Biosciences) overnight at 4°C. The following day, the blot was probed simultaneously with serum from a rabbit immunized with E2wt (1∶8000) and the human mAbs 2F5 or 4E10 (1∶20,000) for 1 h at room temperature. Primary Abs were prepared in Odyssey Blocking Buffer 1∶1 with 1xPBS, 0.2% Tween-20. Blots were washed 5 times with 0.1% Triton X-100, 1xPBS. Secondary Abs IRDye 680 Goat anti-Rabbit and IRDye 800CW Goat anti-Human (LI-COR Biosciences) were used at 1∶15,000, diluted in Odyssey Blocking Buffer 1∶1 with 1× PBS, 0.2% Tween-20, 0.02% SDS. Membranes were scanned using the LI-COR Odyssey Infrared Imaging System (LI-COR Biosciences) to allow simultaneous two-color detection of E2 and the HIV-1 Envelope 2F5 or 4E10 epitopes. Integrated intensities were used in conjunction with protein concentrations determined by NanoDrop (NanoDrop Technologies, Wilmington DE) to calculate protein purity and concentration.

### Biosensor Analyses

Surface plasmon resonance (SPR) biosensor assays were carried out at 25°C using the Biacore T200 instrument (GE Healthcare, Piscataway, NJ). For all experiments, 10 µg/ml of E2-wt, Env(V3)-E2 [39] or Env(MPER)-E2 particles were diluted in sodium acetate buffer (pH 5.5) and immobilized by standard amine coupling chemistry on a CM3 sensor chip. Env(V3)-E2 and Env(MPER)-E2 particles were immobilized to a level of 100 RU on flow cell 3 and 4, respectively. To increase our ability to detect binding to the E2 scaffold, E2wt (lacking the HIV N-terminal fusion) was immobilized in flow cell 2 to approximately six times the level of Env(V3)-E2 and Env(MPER)-E2 (580 RU). Flow cell 1 was activated and blocked and its response was subtracted from all other flow cells. Binding experiments were carried out by injecting the monoclonal Abs 2F5, 4E10, or 10E8 over the sensor surface at concentrations of 0 nM, 18.75 nM, 75 nM and 300 nM in HBS-EP buffer (10 mM HEPES, 150 mM NaCl, 1 mM EDTA, 0.05% P20) at a flow rate of 30 µl/min for 5 minutes (association phase). Following a 10-minute dissociation phase, the chip surface was regenerated for each concentration of injected Ab with a 45 second pulse of 10 mM glycine, pH 2.5. All data were double reference subtracted with buffer blank injections. Data were processed using T200 evaluation software.

### Construction of HIV-1 Env immunogens lacking the variable loop regions

The introduction of deletions within the V2 (ΔV2), V3 (ΔV3), and V1V2 (ΔV12) loops of SF162 were previously reported, where identical residues were omitted as described in [44]. The SF162 ΔV2 protein was generated by deleting 90 nucleotides encoding 30 amino acids from Threonine (T) at position 160 to Tyrosine (Y) at position 189, and the ΔV3 protein omitted 21 amino acids from T 301 to Glycine (G) at position 321. The SF162 ΔV1V2V3 deletion was constructed by first eliminating the combined V1V2 loops (nucleotides encoding 66 amino acids within the V1V2 region from T 127 to Isoleucine (I) at position 192), and then introducing the V3 loop deletion (from T301-G321) into the same construct. Each deletion was replaced with a GAG peptide as reported previously [44], [45]. Although the same residues were omitted as described previously [46], a different mutagenesis strategy was used. Briefly, the Quikchange Lightning Site-directed Mutagenesis Kit (Strategene) was used to mutagenize the pEMC* plasmid (a derivative of p1A* [47]) harboring the SF162 gp160 *env* sequence using the following primers:

SK21(V2) AACTGCAGCTTCAAGGTG**GGCGCCGGC**AAGCTGATCAACTGCAAC;

SK22(V2) GTTGCAGTTGATCAGCTT**GCCGGCGCC**CACCTTGAAGCTGCAGTT;

SK25(V3) GCCCCAACAACAAC**GGCGCCGGC**GACATCCGCCAGGC;

SK26(V3) GCCTGGCGGATGTC**GCCGGCGCC**GTTGTTGTTGGGGC;

SK29(V1V2) TGACCCCCCTGTGCGTG**GGCGCCGG**CAACTGCAACACCAG; and

SK30(V1V2) CTGGTGTTGCAGTT**GCCGGCGCC**CACGCACAGGGGGGTCA. These primers replaced the encoded variable loop with an encoded Gly-Ala-Gly peptide sequence, highlighted in bold. Plasmids encoding the *env* lacking the respective variable loops were transformed into XL-10 Gold Ultracompetent Cells, and screened via PCR. Mutations were confirmed by sequencing. The variant loop Env proteins were expressed on the cell surface, as confirmed by immunofluorescence using COS-7 cells (S1 Figure). These variant loop Env proteins were shown to bind recombinant soluble CD4 in ELISA assays, but pseudoviruses constructed using these plasmids were noninfectious in TZM-bl cells (data not shown).

### Preparation of Helios Gene Gun DNA gold bullets

Plasmid DNA expressing codon-optimized SF162 gp160 and variable loop deletion variants were precipitated onto 1 µm diameter gold beads, and bullets were prepared according to the manufacturer's instructions (Bio-Rad). Each bullet was loaded with a total of 2 µg of DNA. To verify that the bullets were functional, COS-7 cells were transfected with the DNA carried by the gold beads, as described previously [48]. Cells were incubated at 37°C for 48 h, fixed, and then stained for immunofluorescence using 0.5 µg/mL IgG purified from an HIV-1 infected chimpanzee (CHIVIG [49]) as a primary Ab and a 1∶50 dilution of the FITC-conjugated goat anti-human IgG (Zymed) secondary Ab. The presence of envelope-transfected cells was visualized by fluorescent microscopy.

### Purification of serum IgG

IgG from individual rabbit sera from weeks 6, 14, and 28 was purified by affinity chromatography as previously described [48]. For each sample, IgG elution fractions with the highest protein concentration were pooled and dialyzed against 1× PBS at 4°C in SnakeSkin Dialysis Tubing with a MWCO of 10 kDa (Thermo Fisher Scientific). Purity was assessed by SDS-PAGE using the LI-COR Odyssey system as described above and was found to be equal to or greater than 90%. IgG purified from a pool of pre-immune sera and sera from rabbits inoculated with E2wt only or SF162 DNA only were used a negative controls in ELISA and neutralization assays.

### Enzyme-linked immunosorbent assay (ELISA)

Binding Ab responses from individual rabbits to the E2wt protein, HIV-1 Clade B Envelope antigen gp140, gp41 ΔTM (BaL, Immune-tech), and MPER and V3 linear peptides were measured by ELISA as described previously [38]. Endpoint titers were calculated as the lowest positive value for each sample that was three times the average background of pre-immune rabbit serum included in triplicate on each plate. Binding Ab responses to overlapping 15-mer linear epitopes of gp160 (Consensus B) were also measured using ELISA. Individual peptides were added (0.05 ml at 0.01 mg/ml) to flat-bottom 96-well plates (Thermo Scientific), and then the plates were incubated overnight at 4°C. Plates were blocked with Blotto (PBS, 5% nonfat dry milk, 1% goat serum) for 2 hours. Samples were pooled by group for each time point and tested at a dilution of 1∶25 against each peptide. Serum was then incubated for 1 hour at room temperature, and then washed five times with Wash Buffer (PBS, 0.1% Trition X-100). Goat anti-rabbit IgG-HRP at 1∶4000 in Disruption Buffer (PBS, 5% FBS, 2% BSA, 1% Trition X-100) was then added to the plate and incubated for one hour at room temperature. The plates were washed, and TMB substrate (Sigma) was added to each well and incubated for 30 minutes in the dark. The reaction was stopped with 1N H_2_SO_4_, and the plates were read at 450 nm (Molecular Devices SpectraMax 190). Optical density (OD) values were calculated for each group by subtracting two times the average background of pre-immune rabbit serum included in triplicate on each plate. Because each region has a different number of peptides due to size differences, these responses were normalized to compare responses between each HIV Env region. Normalized values reported for each region are the sum of responses (OD values minus background) divided by the number of peptides per region.

### Pseudovirus generation and determination of virus titers

Production of pseudoviruses was described previously [50]. Briefly, pseudoviruses were produced using the pSG3ΔEnv DNA plasmid encoding the HIV backbone and a plasmid encoding either the homologous SF162 immunogen or heterologous BaL.26, SS1196.1, ZM109.PB4, JRCSF, JRFL, YU2 envelope variants. HIV-1 clones JRCSF and JRFL were provided by D. Burton. The following reagents were obtained through the NIH AIDS Research and Reference Reagent Program, Division of AIDS, NIAID, NIH: HIV-1 clone BaL.26 (Cat #11446) from J. Mascola; HIV-1 clone SS1196.1 (Cat #11020) from D. Montefiori and F. Gao; and HIV-1 clone ZM109F.B4 (Cat #11314) from C.A. Derdeyn and E. Hunter. The viral backbone plasmid pSG3ΔEnv was provided by J. Overbaugh [51]. For neutralization assays, the amount of 200 50% tissue culture infective doses (TCID_50_) was calculated according to the method of Reed and Muench [52].

### Production of HIV-2/HIV-1 MPER chimeric virus stocks

The HIV-2/HIV-1 chimeric viruses 7312A (wt), 7312A C1 (MPER), 7312A C3 (2F5) and 7312A C4 (4E10) were provided by G. Shaw [53]. Chimeric viruses were produced by transfecting 293T cells with the infectious molecular clone DNA. Transfected cells were allowed to incubate for 48 hrs at 37°C, 5% CO_2_. Supernatants were collected and centrifuged for 5 minutes at 3,000 rpm to remove cellular debris. The collected virus was aliquoted and stored at -80°C until needed. Virus titers were obtained as described above.

### Neutralization assays

Serum and purified IgG samples were tested for their ability to neutralize HIV-1 Env-containing pseudoviruses using the single-cycle TZM-bl neutralization assay as described previously [50], [54]. Neutralization activity of each sample was determined on the basis of the reduction in the *luc* reporter gene expression compared to that obtained in virus control wells containing virus and cells only, such that {[((virus+cells)-background)-((serum+virus+cells)-background)]/(virus+cells)-background} ×100 = % neutralization. Background control wells contained cells only; all samples were tested twice in duplicate. As a negative control, a pool of the pre-immune serum was used and the lowest dilution tested (1∶8) never achieved 50% neutralization. Neutralization dose-response curves were fitted by non-linear regression and a final titer is reported as the reciprocal of the dilution of serum necessary to achieve 50% neutralization. For mAbs and purified IgG, the concentration of Ab required to obtain 50% neutralization (the 50% inhibitory concentration [IC_50_]) is reported.

Detection of NAbs specifically directed to V3 and MPER peptides was performed as previously described [55]. Briefly, the TZM-bl neutralization assay was conducted with the inclusion of HIV-1 SF162 V3, MPER, or scrambled peptides for 1 h, at a final concentration of 20 µg/mL with titrated rabbit sera or purified IgG (week 14, 22, and 31) prior to 1 h incubation with 200 TCID_50_ of SF162 or 7312A C1 HIV-2/HIV-1 chimera pseuoviruses. Percent change in neutralization (IC_50_) was calculated as: [(IC_50_ without peptide)-(IC_50_ with peptide)/(IC_50_ without peptide)] ×100.

### Antibodies and synthetic peptides

MAbs 2F5, 4E10, and 10E8 to HIV-1 MPER were obtained through the NIH AIDS Research and Reference Reagent Program, Division of AIDS, NIAID, NIH and purchased from Polymun Scientific (Vienna, Austria). For peptide competition assays and ELISAs, the HIV-1 SF162 V3 peptide (PNNNTRKSITIGPGRAFYATGD), MPER peptide (RRRNEQELLELDKWASLWNWFDITNWLWYIRRRR), MPER N-terminal peptide (NEQELLELDKWASLWN), MPER C-terminal peptide (KFWDKWILSWINIWYLFY) and MPER scrambled peptides (KKKRIYWLWNTIDFWNWLSAWKDLELLEQENKKK) and (WEALNLQLKLNWESDE) were synthesized by Genscript (Piscataway, NJ) with a purity >80%. Consensus B gp160 peptides and MPER derivatives: 162 (SQNQQEKNEQELLEL), 163 (QEKNEQELLELDKWA), 164 (EQELLELDKWASLWN) used for epitope mapping were obtained through the NIH AIDS Research and Reference Reagent Program.

### Statistics

Statistical analyses were performed using either the unpaired Student t-test or the repeated measures ANOVA test to compare data sets, with a *P*-value obtained for all comparisons. Differences were considered statistically significant when *P*<0.05, as represented by “*”, *P*<0.01, as represented by “**”, or *P*<0.001, as represented by “***”. When comparisons are statistically significant, *P*-values are indicated in the figure legends. For binding and neutralization assays, the results are expressed as the average +/− SEM, unless specified otherwise in the figure legend.

## Results

### Construction and purification of multimeric scaffold particles displaying the gp41 extracellular domain and MPER

Nucleotides encoding the HIV-1 SF162 extracellular domain of gp41 (aa 513–678 HXB2 numbering; 165 aa total) were ligated in frame to the 5′ end of the linker upstream to the *E2* gene within the plasmid pE2DISP, resulting in an N-terminal fusion of the gp41 ectodomain to the E2 protein (referred to as ‘Env(gp41)-E2’ for the remainder of this manuscript) (Fig. 1A). To focus responses directly to MPER, nucleotides of SF162*env* encoding aa 648–689 (41 aa total) were also ligated in frame to the 5′ end of the *E2* gene, resulting in an N-terminal fusion of MPER to the E2 protein (referred to as ‘Env(MPER)-E2’ for the remainder of the manuscript) (Fig. 1A). These resulting N-terminal fusion proteins were thus displayed without constraint on the surface of the 60-mer particles. Env(MPER)-E2 and Env(gp41)-E2 were expressed in *E. coli*, and purified from inclusion bodies. The solubility and stability of these particles substantially increased when they were refolded in the presence of the E2wt core protein, with no observed precipitation. Therefore, solubilized fusion proteins were refolded in the presence of E2wt core protein (E2 monomers without the N-terminal HIV-1 fusion) in step-down dialysis. Env(MPER)-E2 was refolded with equimolar amounts of E2wt, and Env(gp41)-E2 required a 1∶3 ratio of Env(gp41)-E2 fusion protein:E2wt to remain fully soluble.

The final refolded 60-mer particles were purified by gel filtration, which resulted in a single peak in the void volume (Fig. 1B). While size separation cannot exclude differently sized particles, the transmission electron microscopy (TEM) demonstrated the integrity, symmetry, and purity of the dominant species at the correct size (Fig. 1C). Both Env(gp41)-E2 and Env(MPER)-E2 were of similar size and shape compared to E2wt particles illustrated in previous studies [39], [40]. Resulting multimeric particles were>90% pure as determined by quantitative analysis of the SimplyBlue-stained protein gels run under reducing conditions (Fig. 1D, left panel). Identity of these particles was assessed by Western blot analysis using the LI-COR Odyssey system, which allows sensitive and simultaneous detection of HIV-1 Env and E2 components within the same blot (Fig. 1D, right panels). Both constructs contained the epitope recognized by mAb 2F5, and only the Env(MPER)-E2 construct contained the epitope recognized by the 4E10 epitope (Fig 1A, D). The Env(gp41)-E2 construct lacked residues leucine and tryptophan at positions 679 and 680, respectively (Fig. 1A), which accounted for only a partial 4E10 epitope that was not recognized by the mAb 4E10 (Fig. 1D).

Surface plasmon resonance (SPR) was used to determine the availability of the MPER on the surface of the Env(MPER)-E2 particles. Binding of the 2F5 NmAb to the Env(MPER)-E2 particles was detected by SPR, indicating that the 2F5 epitope was accessible on the surface of the Env(MPER)-E2 particles (Fig. 1E). No binding of the 4E10 NmAb was detected to the Env(MPER)-E2 particles, although this construct contained the 4E10 epitope, suggesting that the 4E10 epitope was inaccessible on the surface of the Env(MPER)-E2 particles (data not shown). Likewise, no binding of the Env(MPER)-E2 particles was detected to the 10E8 NmAb, which has a partial overlapping binding site to the 4E10 epitope. The E2wt scaffold and Env(V3)-E2 particles served as negative controls, and no binding of 2F5, 4E10, or 10E8 NmAbs was detected to either of these particles, as expected (Fig. 1E). An empirical Kd value of the 2F5 mAb to the Env(MPER)-E2 particle could not be accurately obtained due to the multivalency of the MPER peptide on the surface of the particle and the bivalency of the 2F5 mAb. Long off rates were observed and most likely due to the overall avidity of these interactions.

### Co-immunization with Env(MPER)-E2 and Env(gp41)-E2 multimeric scaffolds and gp160 DNA induced HIV-1 specific binding antibodies

**Figure 2:**
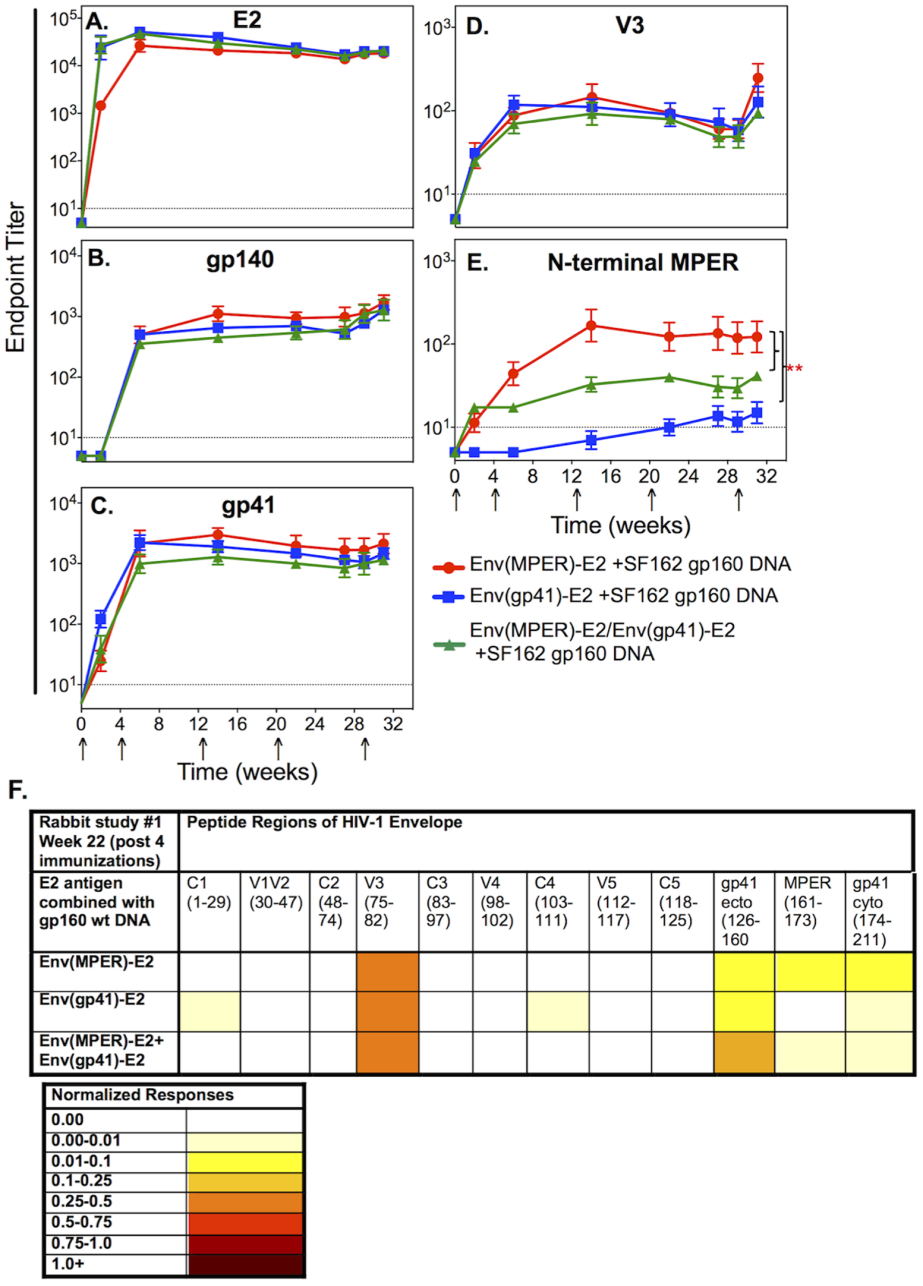
Binding antibody responses elicited in rabbits co-immunized with Env(MPER)-E2 and/or Env(gp41)-E2 multimeric particles and SF162 gp160 DNA. Binding Ab responses to the (A) E2 scaffold protein, (B) Clade B gp140, (C) Clade B gp41, (D) SF162 V3 peptide, and (E) SF162 N-terminal MPER peptide were determined by ELISA. Values graphed are the geometric mean (+/− SEM) endpoint titers for individual rabbits in each of the three groups (highlighted in red, blue and green) over the course of the experiment. Stars indicate significant binding of Abs to the N-terminal MPER peptide elicited in the Env(MPER)-E2 group compared to the Env(gp41)-E2 group (*P*<0.001) and the Env(MPER)-E2/Env(gp41)-E2 group (*P*<0.01) as determined by repeated measures ANOVA analysis. Pre-immune sera and sera from a rabbit inoculated with the E2wt scaffold were used as negative controls. The dashed line on each graph represents three times the average background of these negative controls included in triplicate on each plate. (F) Binding Ab responses to overlapping peptides spanning the Clade B gp160 Env were also measured using ELISA at week 22 at a dilution of 1∶25. In order to compare responses between each HIV Env region, responses were normalized for each group by the summation of OD values per region minus background, divided by the number of peptides per region. Darker colors indicate increased binding to each region listed at the top of each column.

Previous studies in our lab using the E2 multimeric scaffold displaying the third hypervariable loop (V3) of Env revealed that Ab responses significantly increased when rabbits were co-immunized with the Env(V3)-E2 particles and HIV-1 gp160 plasmid DNA, compared to protein alone or DNA prime, protein boost [39]. Therefore, we tested the ability of Env(MPER)-E2 and Env(gp41)-E2 multimeric particles, in combination with HIV-1 SF162 codon-optimized gp160 DNA, to generate humoral responses in New Zealand white rabbits. Group 1 received Env(MPER)-E2 particles (200 µg, IM) co-immunized with gp160 DNA (36 µg per dose, ID). Group 2 was immunized with particles displaying gp41 [Env(gp41)-E2] plus gp160 DNA at the same doses in Group 1. Group 3 (mixture of MPER+gp41 particles plus gp160 DNA) received 100 µg each of (Env(MPER)-E2 and Env(gp41)-E2). Rabbits were immunized at weeks 0, 4, 12, 20, and 29. Blood draws were collected two weeks after each immunization as well as at weeks 27 and 29 and each sample from each rabbit was analyzed for binding to E2, gp140, gp41, the V3 peptide and the N-terminal MPER peptide. All rabbits generated high titers of E2-specific Abs after the first immunization with maximal responses achieved following the second vaccination (Fig. 2A), which were sustained throughout the course of the experiment in all rabbits. There was no significant difference between the groups in the potency of E2 binding Abs, suggesting delivery of these particles was equivalent in all groups. Similarly, each vaccine regimen elicited equivalent levels of gp140- and gp41-specific binding Abs after two inoculations that were maintained throughout the course of the experiment (Fig. 2B, C).

### Envelope epitopes were recognized following co-immunization with Env(MPER)-E2 and Env(gp41)-E2 multimeric scaffolds and gp160 DNA

Individual rabbit Ab responses were further analyzed longitudinally by measuring responses to the V3 peptide and to an N-terminal MPER peptide overlapping the 2F5 mAb epitope (Fig. 2D, E). All rabbits in all groups had high titers of binding Abs specific for V3 (present only in the gp160 DNA immunogen) following the first vaccination. These responses subsequently increased and reached maximum values following the second vaccination (Fig. 2D), decreased slightly when immunizations were abstained after week 20, and then returned to maximum levels after immunizing at week 29 (Fig. 2D). Despite similar binding Ab responses to gp140 and gp41, Ab responses to MPER varied among the groups. The Env(MPER)-E2 group had significantly higher binding Abs to the N-terminal MPER peptide compared to either the Env(gp41)-E2 (*P*<0.001) or Env(MPER)-E2+Env(gp41)-E2 (*P*<0.01) groups (Fig. 2E). Minimal binding Ab responses were also detected to a C-terminal MPER peptide overlapping the 4E10 epitope, with no difference between the groups (data not shown). To characterize the specificity of the binding Ab response to Env overall, we further analyzed pooled sera at week 22 by peptide ELISA using overlapping peptides spanning the Clade B consensus gp160 Env (Fig. 2F). Strong binding to the V3 peptide was observed in all groups, and binding to MPER was seen only in rabbits that received Env(MPER)-E2 particles (Fig. 2F).

### Env(MPER)-E2 and Env(gp41)-E2 particle vaccines elicited autologous NAbs dominated by V3 loop specificity

**Figure 3:**
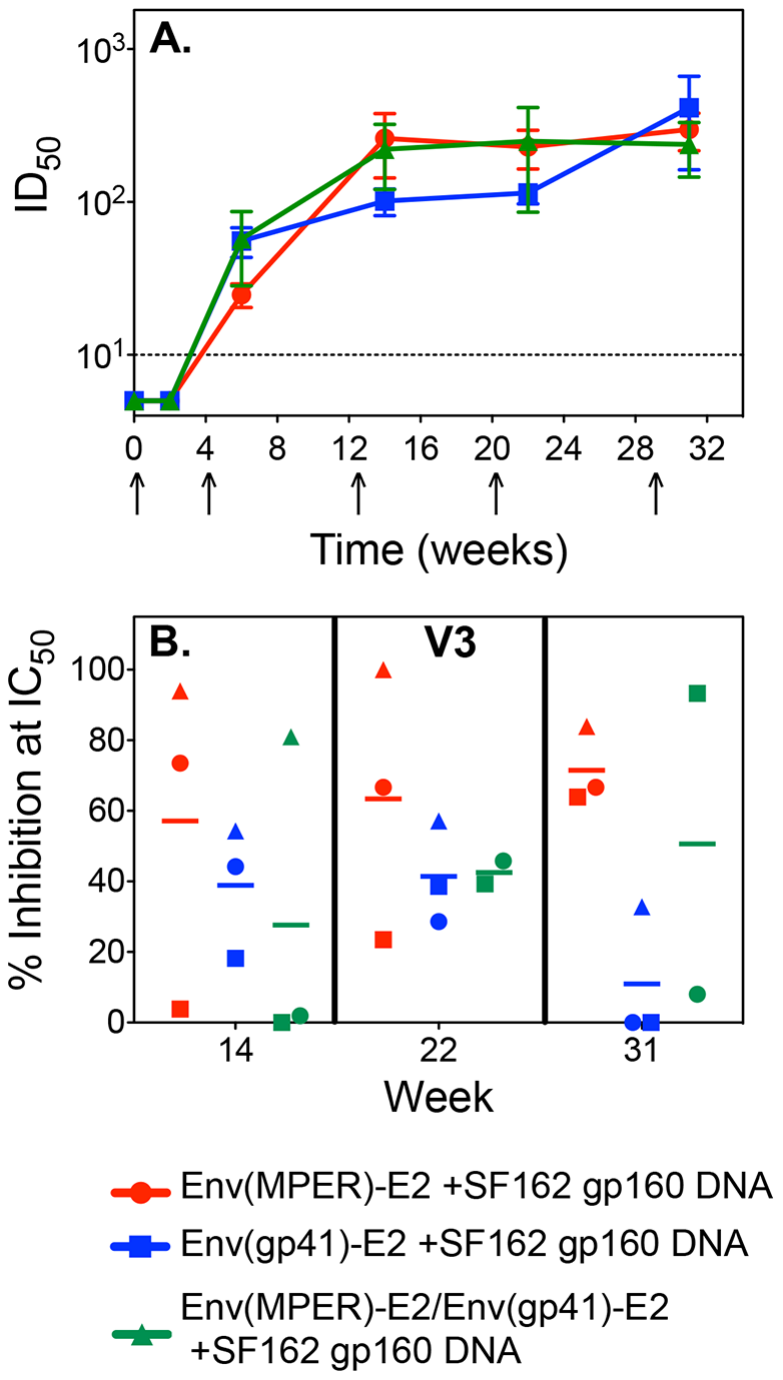
Specificity of the neutralizing antibody responses to SF162 elicited in rabbits co-immunized with Env(MPER)-E2 and/or Env(gp41)-E2 multimeric particles and SF162 gp160 DNA. (A) NAb titers were determined by TZM-bl pseudovirus assays against the homologous SF162 Env. Values graphed are the mean ID_50_ values for individual rabbits in each of the three groups (highlighted in red, blue and green) over the course of the experiment. The dashed line represents the assay background, as determined by pre-immune sera and sera from a rabbit inoculated with the E2wt scaffold. (B) The contribution of V3-specific NAbs, was determined by peptide neutralization inhibition assays. Samples from individual rabbits at two weeks after the third (week 14), the fourth (week 22), and the final immunizations (week 31) were analyzed. Data are graphed as the individual responses of each rabbit with the mean percent of neutralizing responses inhibited by the V3 peptide for each group.

Individual rabbit sera were analyzed for neutralizing activity against the autologous HIV-1 subtype B isolate SF162 (Tier 1) in the single round replication TZM-bl neutralization assay and there was very close agreement in all responses in each group. SF162 NAbs were detected after two immunizations in each group, and these titers reached maximum levels following the third vaccination (Fig. 3A). As an additional negative control, serum from a single rabbit immunized with the E2wt scaffold (non recombinant) was tested, and no neutralizing activity against SF162 was detected (data not shown). There was no significant difference in the levels of NAb titers between the groups throughout the course of the experiment. To determine the specificity of the NAbs, competition studies were conducted using a matched V3 peptide. These studies revealed that NAbs specific to V3 dominated the response in the majority of the rabbits immunized with Env(MPER)-2 particles and gp160DNA at weeks 14, 22, and 31 after three, four, or five immunizations, respectively (Fig. 3B). Interestingly, there were fewer V3 specific NAbs elicited in rabbits immunized with Env(gp41)-E2 and gp160 DNA at week 31, as noted by no inhibition with the V3 peptide in two of the three rabbits after five immunizations.

Two different approaches were used to determine if MPER-specific NAbs were elicited in this rabbit experiment. In the first approach, purification of MPER-specific Abs from rabbit serum was tested using an MPER-peptide affinity column. Minimal neutralization activity was detected in the elution fractions from each group, however non-specific Ab binding to the column was also observed (data not shown). After multiple attempts, rabbit serum samples were exhausted due to limitation in blood draw volumes. We concluded that the MPER-specific Abs were too low in concentration to obtain reliable results from this experiment. In a second approach, we employed peptide inhibition of neutralization using full-length and N-terminal MPER peptides, and we only observed modest (10%) reduction in NAb titers (data not shown). Therefore, from these sets of experiments we could not delineate if MPER-specific NAbs were elicited using the Env(MPER)-E2 particles co-immunized with DNA.

### Deletion of variable loops within the gp160 DNA component of the vaccine redirects antibody responses to MPER when co-immunized with Env(MPER)-E2 particles

Previous studies have suggested that removal of the V1, V2, or V3 loops may redirect immune responses towards other neutralization determinants [56]–[58]. In addition, prior studies have suggested that removal of the V2 loop enhanced the neutralization susceptibility of SF162 to the gp41 mAbs 2F5 and 4E10 [56], [59]. Therefore, in order to redirect the NAb responses away from the V3 region and towards the MPER region, we developed gp160 DNA constructs eliminating selected variable regions. The four new constructs were SF162 full-length gp160 (gp160wt), gp160 with removal of the V3 loop (ΔV3), the V2 loop (ΔV2), or the combined V1, V2, and V3 loops (ΔV123). All constructs expressed similar levels of Env on the surface of transfected cells (S1 Figure), and were capable of binding to sCD4 (data not shown).

**Figure 4:**
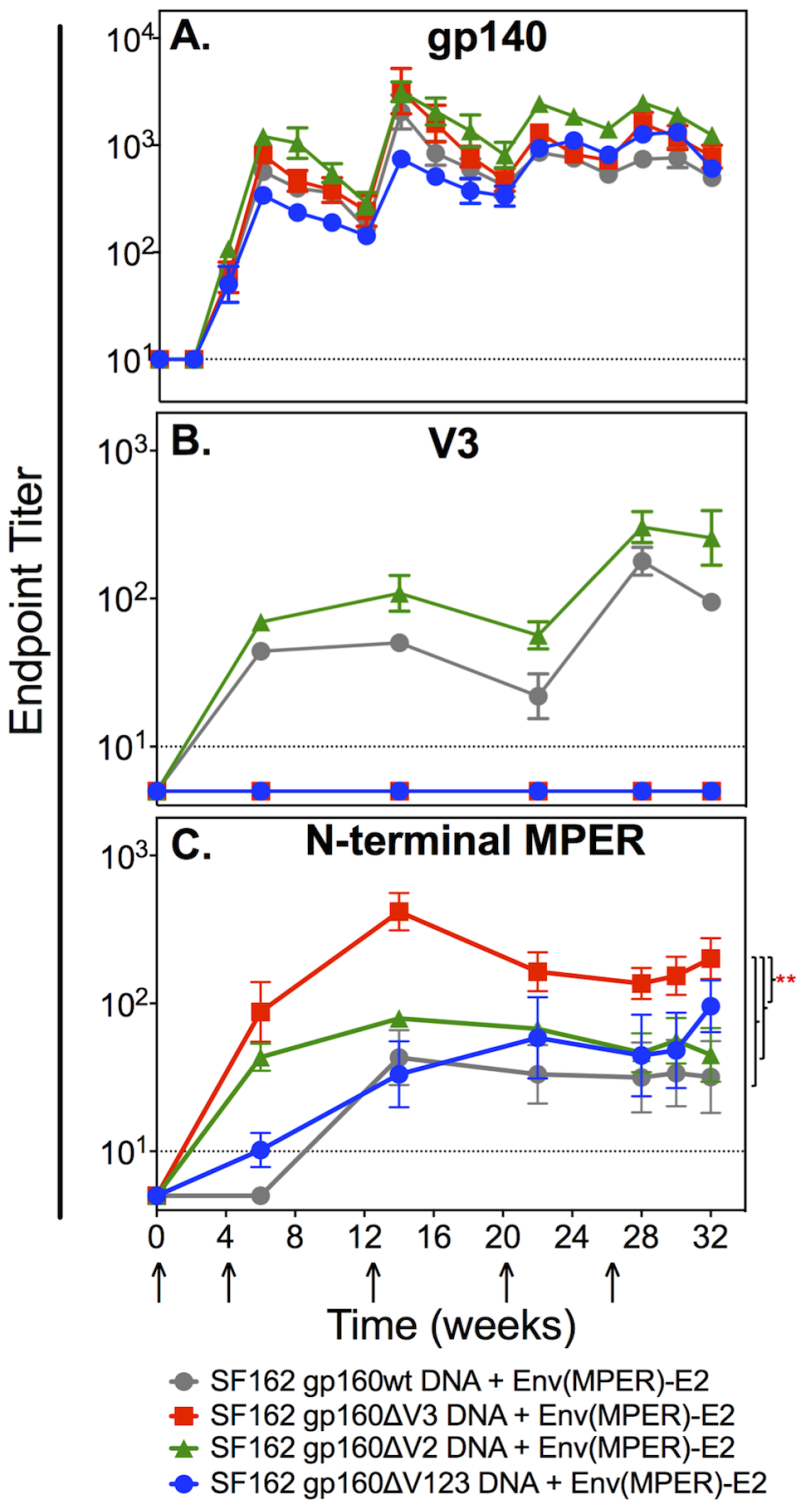
Binding antibody responses in rabbits co-immunized with Env(MPER)-E2 and either SF162 gp160full-length, ΔV3, ΔV2, or ΔV123 DNA. Binding Ab responses to (A) SF162 gp140, (B) V3 peptide, and (C) N-terminal MPER peptide were determined by ELISA. Values graphed are the geometric mean (+/− SEM) endpoint titers of individual rabbits for each of the four groups (highlighted in grey, red, green, and blue) over the course of the experiment. Rabbits immunized with Env(MPER)-E2 particles and ΔV3 gp160 DNA elicited significantly higher N-terminal MPER peptide specific binding Ab responses compared to the other groups (*P*<0.01) as determined by repeated measures ANOVA analysis. Pre-immune sera and sera from a rabbit inoculated with the E2wt scaffold were used as negative controls. The dashed line on each graph represents three times the average background of these negative controls included in triplicate on each plate.

We thus designed a comparative immunization study in rabbits to evaluate the effect of removing these variable regions from the DNA component, and the Env(MPER)-E2 immunogen was chosen as the protein co-immunization partner based upon its ability to elicit Abs targeting MPER (Fig. 2E, 2F, 3C). Sixteen rabbits were divided into four equal groups, each receiving one Env DNA construct co-immunized with Env(MPER)-E2 particles at weeks 0, 4, 12, 20, and 26. The groups are referred to by the DNA construct used (gp160wt, ΔV2, ΔV3, or ΔV123) throughout the remainder of the manuscript. Rabbits were bled every two weeks throughout the course of the experiment to determine the sustainability of the Ab responses. Similar to observations in the first rabbit study comparing Env(MPER)-E2 and Env(gp41)-E2, binding Abs directed to gp140 were detected following the second vaccination (Fig. 4A), based on analyses of individual rabbit sera and graphed as groups. These responses decreased between immunizations, reached maximum titers following the third vaccination, and were stable following additional immunizations (Fig. 4A). Additional immunizations increased the sustainability of these responses between boosting (Fig. 4A). Gp140-specific Ab titers were not statistically different between groups.

Binding Abs directed to V3 peptide were only observed in the sera from the gp160wt and gp160ΔV2 groups, as expected (Fig. 4B). Notably, there was a significant increase in the titers of MPER peptide-specific Abs in the gp160ΔV3 group compared to the other three groups (*P*<0.01) (Fig. 4C). However, there was no significant difference between the MPER-specific titers elicited in the gp160wt, gp160ΔV2 or gp160ΔV123 groups (Fig. 4C).

### Epitope mapping reveals differential targeting of antibody responses

**Figure 5:**
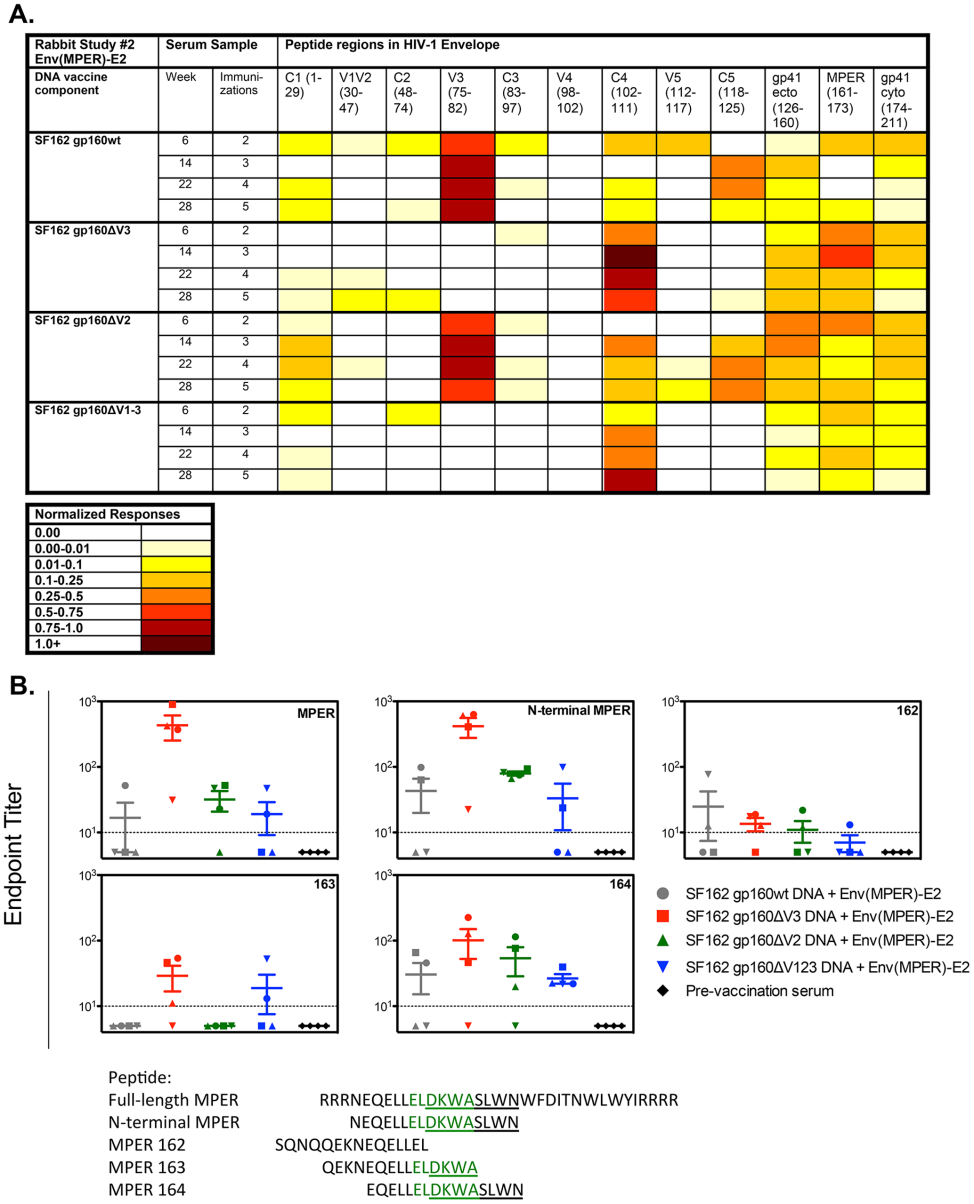
Differential targeting and epitope mapping of binding antibody responses elicited in rabbits co-immunized with Env(MPER)-E2 and either SF162 gp160full-length, ΔV3, ΔV2, or ΔV123 DNA. (A) Serum binding Ab responses to overlapping peptides (15-mers with 11 amino acid overlap) spanning the Clade B gp160 Env were measured by ELISA at weeks 6, 14, 22, and 28. Responses were normalized for each group by the summation of OD values per region minus the background divided by the number of peptides per region. Darker colors indicate increased binding to particular regions listed at the top of the figure. (B) Epitope mapping of individual rabbit serum samples from each group elicited at week 14 to the various MPER peptides listed below the table. The 2F5 mAb epitope is highlighted in green, and underlined is the serine at position 667 required for full MPER binding by the serum samples from each rabbit group. Weak binding by the gp160ΔV3 and gp160ΔV123 groups was observed to MPER peptide 163, indicating that the DKWA may be important for binding. Serum from rabbits immunized with SF162 DNA only and pre-vaccination samples served as negative controls, while the 2F5 mAb served as a positive control.

Similar to the first study, pooled sera were further analyzed by peptide ELISAs using overlapping linear peptides spanning the gp160 Env in order to determine how removal of the variable loops from gp160 affected Ab targeting outside of the V3 and MPER epitopes. Furthermore, to investigate the effects of boosting on temporal changes in Ab targeting, pooled rabbit sera were tested at multiple time points post-vaccination: weeks 6, 14, 22, and 28 (Fig. 5A, S2 Figure). As shown by V3 peptide ELISA (Fig. 4B), V3-specific Abs in the peptide scan were detected only in the gp160wt and gp160ΔV2-immunized rabbits (Fig. 5A). The removal of the V3 region in the gp160ΔV3 and gp160ΔV123-immunized rabbits resulted in a redirection of the Ab responses to C4, as demonstrated by an increased response to this region compared to the other two groups. Responses to C4 increased with additional immunizations in rabbits within the gp160ΔV123 group (Fig. 5A). Of note, there is also a loss of C5 responses in the groups lacking V3 (gp160ΔV3 and gp160ΔV123) compared to the gp160wt group. Ab responses directed to the ectodomain and intracellular domain of gp41 were detected in each group, with the greatest response observed in rabbits immunized with two or three vaccinations of gp160ΔV2 DNA and Env(MPER)-E2 particles. MPER was also recognized by serum from most rabbits (all vaccine groups); however, a stronger binding Ab response to MPER was detected in the gp160ΔV3 group, as seen in the N-terminal MPER peptide ELISA analysis (Fig. 4C). Interestingly, the greatest response to the MPER region was at week 6 or 14 for all of the groups, and the response appeared to wane after the addition of the fourth and fifth vaccinations.

Since MPER-specific binding Abs were detected at the highest titer after two (wk 6) or three immunizations (wk 14), further epitope mapping was performed on the individual rabbit serum samples from week 14 using various MPER peptides lacking specific residues (Fig. 5B). In this assay, pooled pre-vaccinated serum was used a negative control, where binding was not observed to any of the MPER peptides, as expected (Fig. 5B). The 2F5 NmAb bound to the MPER peptides containing the 2F5 epitope, and served as a positive control (data not shown). The individual rabbit serum samples from each rabbit group (gp160wt, ΔV2, ΔV3, or ΔV123) bound to the full-length MPER, and to N-terminal MPER and MPER peptide 164. However, only weak binding was detected to the MPER peptide 162 and only a few rabbits in each of the gp160ΔV3 and gp160ΔV123 groups bound weakly to the MPER 163 peptide (Fig. 5B). The majority of the Ab binding is observed in peptides containing the DKWASLWN with strong binding only observed with SLWN containing peptides, suggesting that binding Abs elicited by these rabbits required the C-terminal SLWN region for MPER-specific binding. Importantly, binding to any MPER peptides was not observed using serum from rabbits immunized with SF162 gp160wt DNA only [48], further showing that the Env(MPER)-E2 particles in the vaccine regimen were responsible for eliciting the MPER-specific binding Abs (data not shown).

### Env(MPER)-E2 particles and gp160DNA vaccine-induced activity against HIV-SF162 and HIV-2/HIV-1 MPER chimeras

**Figure 6:**
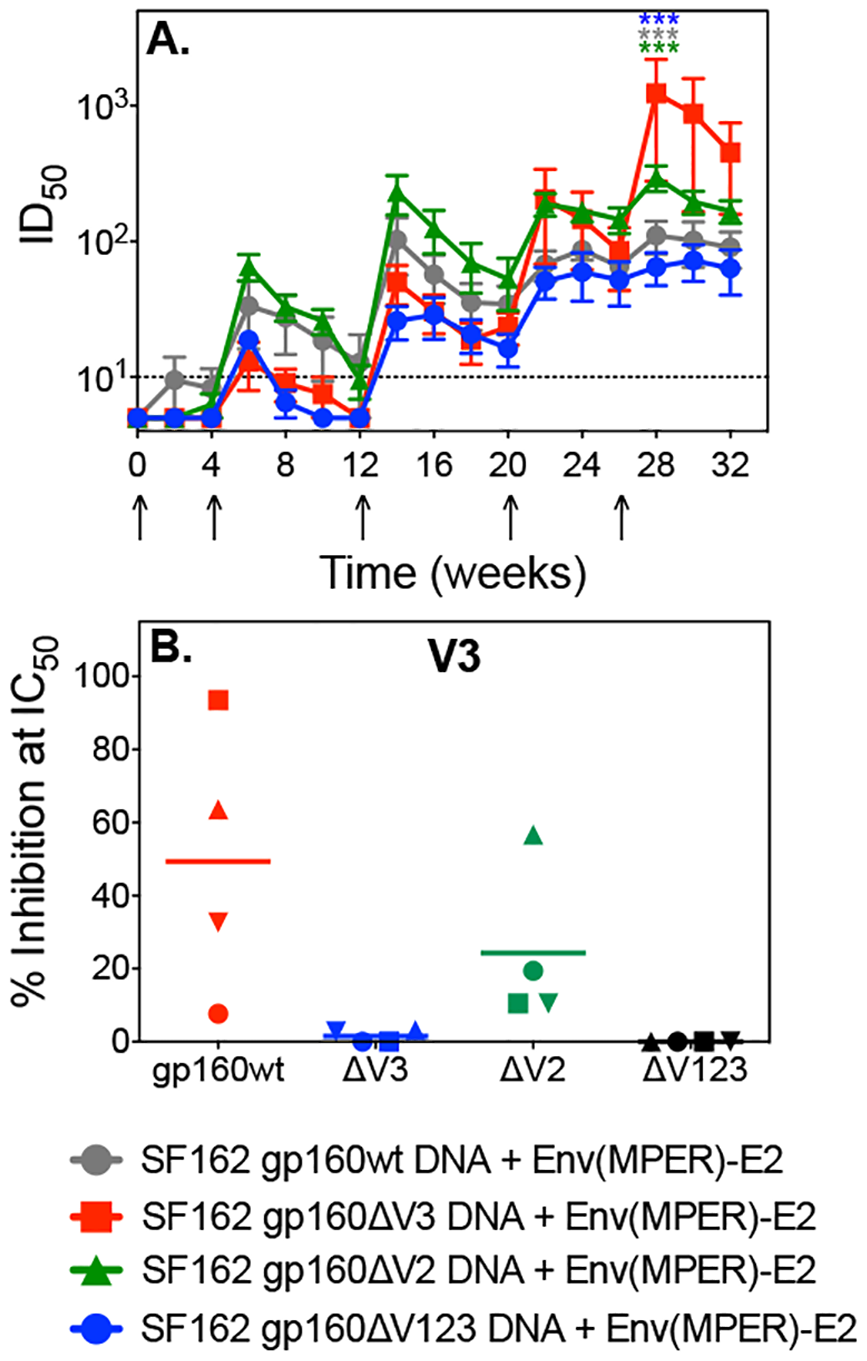
Neutralizing antibody responses to SF162 elicited in rabbits co-immunized with Env(MPER)-E2 and either SF162 gp160full-length, ΔV3, ΔV2, or ΔV123 DNA. (A) Neutralization titers against the autologous SF162 pseudovirus were determined using the TZM-bl assay. Values graphed are the mean ID_50_ values for individual rabbits in each of the four groups (highlighted in grey, red, green, and blue) over the course of the experiment. The dashed line represents the assay background, as determined by pre-immune sera and sera from a rabbit inoculated with the E2wt scaffold. Rabbits immunized with Env(MPER)-E2 particles and gp160ΔV3 DNA elicited significantly higher autologous NAbs compared to other groups (*P*<0.01) after five immunizations (week 28) as determined by repeated measures ANOVA analysis. (B) The contribution of V3-specific neutralization at week 28 was determined by peptide neutralization inhibition assays. Data are graphed as the individual response of each rabbit with a line indicating the mean percent of neutralizing responses inhibited by the V3 peptide for each group.

Sera from each rabbit co-immunized with variant gp160 DNA immunogens and Env(MPER)-E2 particles were tested individually for their ability to neutralize the autologous virus, SF162. All vaccine regimens stimulated similar HIV-SF162 NAb titers following a second vaccination (Fig. 6A). These responses waned and were boosted by the third vaccination before reaching stable levels following the fourth vaccination, trends similar to what was observed in the gp140 binding Ab response (Fig. 4A). However, rabbits co-immunized with Env(MPER)-E2 particles and gp160ΔV3 DNA generated significantly higher autologous NAbs compared to the other groups at week 28 (*P*<0.01) (Fig. 6A). Although all of the groups initially had similar NAb titers, the targets of the NAbs differed between the groups (Fig. 6B). As observed in the first study, the majority of NAb responses elicited by the gp160wt group were directed to V3 (Fig. 3B, 6B). Comparable inhibition was observed in the gp160ΔV2 group by the V3 peptide (Fig. 6B). Neutralization activity of the gp160ΔV3 and gp160ΔV123 groups was not affected by V3 peptide, as expected. Similar to the first study, purification of MPER-specific Abs from rabbit serum was thoroughly examined using an MPER peptide affinity column. Although neutralizing activity was found in the elution fraction, we observed some nonspecific binding to the column. Likewise, only a very small portion (<10%) of the neutralizing activity in any group was inhibited by the MPER peptide. Therefore, we could not delineate from these experiments if the purified Abs were specific to MPER or other neutralization epitopes.

**Figure 7:**
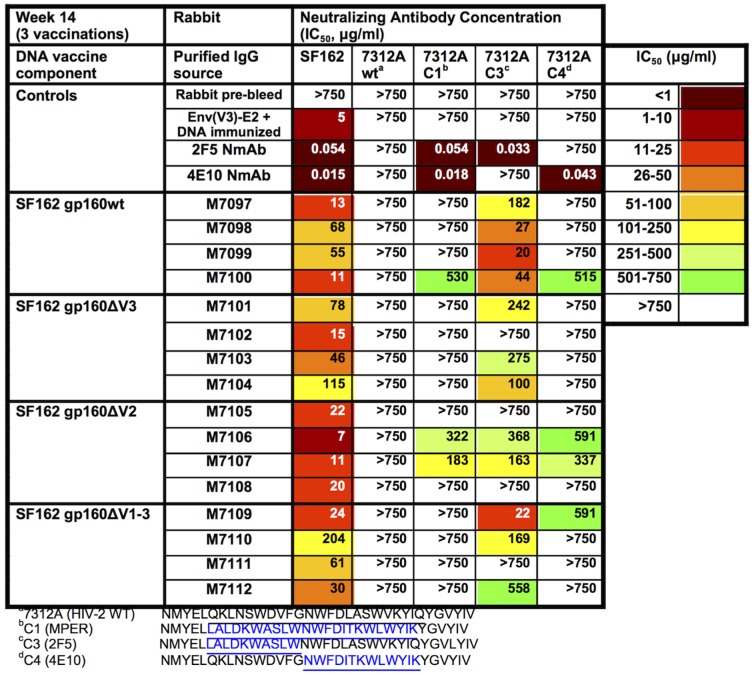
Neutralization Activity Against HIV-2/HIV-1 MPER Chimera Viruses elicited in rabbits co-immunized with Env(MPER)-E2 and either SF162 gp160full-length, ΔV3, ΔV2, or ΔV123 DNA. (A) Neutralization titers against the autologous SF162 pseudovirus, parental HIV-2 virus 7312A wt, and the HIV-2/HIV-1 MPER chimera viruses 7312A C1, C3, and C4 were determined using the TZM-bl assay. Values stated are the IC_50_ values for individual rabbits in each of the four groups at week 14 following the third vaccination. Pre-bleed samples as well as sera from a Env(V3)-E2+SF162 gp160 DNA vaccinated rabbit were included as controls. Darker colors represent an increase in NAb titer.

To test for MPER-specific neutralization, several HIV-2/HIV-1 MPER chimeric Env viruses [53] were used to test purified IgG from individual rabbit sera. No activity directed to the parental HIV-2 7312A virus was observed in any of the test samples or with the control mAbs, 2F5 and 4E10 (Fig. 7). As expected, the 2F5 and 4E10 NmAbs neutralized the chimeric viruses bearing their respective epitopes (Fig. 7). Notably, no MPER-specific NAbs were detected in IgG from rabbits co-immunized with E2 particles displaying the V3 epitope (Env(V3)-E2 particles) and gp160wt DNA, samples generated from a prior published study [60]. However, NAbs to 7312A C3 (HIV-1 2F5 epitope) were observed in 12/16 rabbits that received the Env(MPER)-E2, representing all groups following the third immunization (Fig. 7). All rabbits that received gp160wt DNA and Env(MPER)-E2 particles had NAbs directed to the N-terminal MPER epitope, with a range of 20–182 µg/ml. Three of the four rabbits in the gp160ΔV3 and gp160ΔV123 group developed NAbs directed to the N-terminal MPER epitope with a range of 100–275 µg/ml and 22–558 µg/ml, respectively. Two of the four rabbits in the gp160ΔV2 group developed NAbs directed to the N-terminal MPER epitope with a range of 163–368 µg/ml. Additionally, four rabbits developed NAbs to 7312A C4 (C-terminal MPER), ranging from 337–59 µg/ml (Fig. 7). We tested full length MPER and MPER scrambled peptides for their ability to inhibit neutralization of HIV-2 chimeras C1, C3, C4, and specific competition was less than 10% difference in IC_50_, comparing scrambled with non-scrambled peptides (data not shown). Neutralization by mAbs 4E10 and 2F5 against these viruses (Fig. 7) was fully inhibited by the full length MPER peptide (data not shown).

### Cross-clade neutralizing antibodies were elicited against Tier 1 and 2 viruses

**Figure 8:**
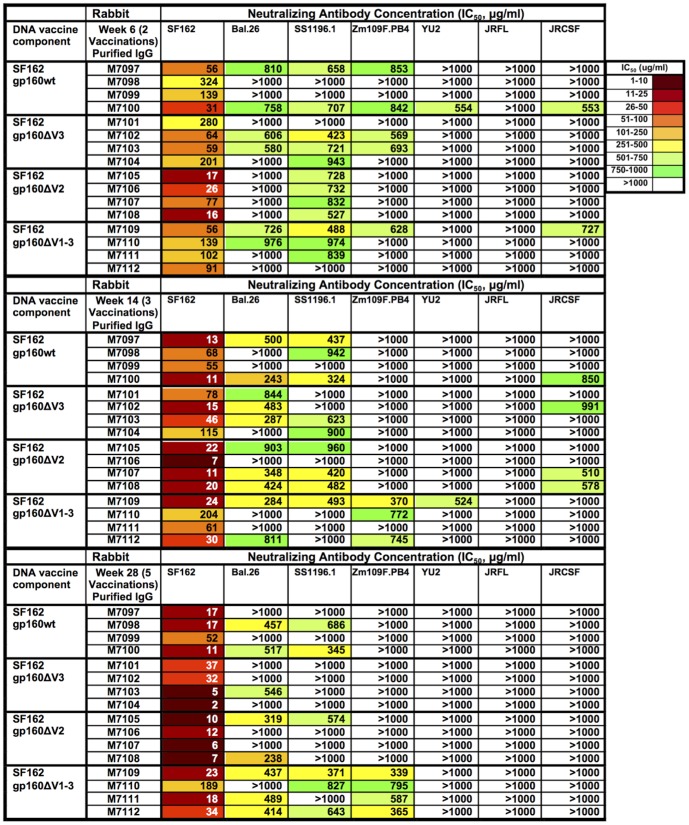
Neutralization Activity Against a Panel of HIV-1 isolates is elicited in rabbits co-immunized with Env(MPER)-E2 and either SF162 gp160full-length, ΔV3, ΔV2, or ΔV123 DNA. (A) Neutralization titers against the autologous SF162, Bal.26, SS1196.1 ZM109F.PB4, YU2, JRFL, and JRCSF pseudoviruses were determined using the TZM-bl assay. Values stated are the IC_50_ values for individual rabbits in each of the four groups at weeks 6 (A), 14 (B), and 28 (C). Darker colors represent an increase in NAb titer.

Purified rabbit IgG from each group was tested for the ability to neutralize pseudotyped Env variants of either HIV-1 Clade B or C to determine how effectively each immunization regimen could elicit cross-NAbs. Five Clade B envelopes [Bal.26 (Tier 1B), SS1196.1 (Tier1B), JRFL (Tier 2), JRCSF (Tier 2), and YU2 (Tier 2)] and one clade C envelope [ZM109F.PB4 (Tier 1B)] were analyzed for neutralization sensitivity using purified IgG from weeks 6, 14, and 28 in the TZM-bl assay. All groups had NAbs against Tier 1 viruses following the second vaccination (wk 6, Fig. 8A). These responses were maintained following the third vaccination (wk14, Fig. 8B), with some additional NAb responses to the Tier 2 virus JRCSF. However, by the end of the vaccination schedule (wk 28), breadth of the NAbs decreased in all groups except the gp160ΔV123 group (Fig. 8C). Three of the strongest responders against multiple HIV-2/HIV-1 MPER chimeras (M7100, M7107, and M7109) also had NAbs in the 251–500 µg/ml range against other HIV-1 isolates, but these did not show strong MPER targeting by peptide ELISA (S3 Figure).

## Discussion

Despite the challenge of sequence variability inherent in the HIV-1 Env, there are a number of conserved regions that are targets of broad NAbs, including the MPER. The goal of this study was to develop a vaccine strategy that induced Abs and NAbs directed specifically to the MPER. Therefore, we rationally designed, expressed, and purified Env-E2 particles displaying the gp41 ectodomain or MPER as unconstrained polypeptides and tested these particles in rabbits in combination with gp160 full-length DNA or deletion variants to determine comparative effectiveness. Here, we provide evidence for the induction of binding Abs directed to the MPER using Env(MPER)-E2 particles co-immunized with gp160 full-length DNA or gp160 DNA lacking variable loops V2, V3, or V123, as well as the induction of NAbs capable of neutralizing several heterologous HIV-1 pseudovirus isolates at modest levels.

Recent vaccine studies have reported advantages to co-immunization with DNA and protein components [61], [62]. The present study expands upon an initial study using E2 particles displaying V3, where co-immunization of Env(V3)-E2 particles with gp160 DNA generated a significant increase in NAbs compared to immunization with protein alone or DNA alone [39]. We elected to utilize this strategy in the current study to compare E2 particles displaying multiple copies of the gp41 extracellular region or the MPER region. Similar to our previous findings, binding Abs and NAbs were detected after two immunizations, and similar levels of binding Abs directed to gp140, gp41 and the V3 peptide were elicited by both of these immunogens. However, only the Env(MPER)-E2 and combined Env(MPER)-E2 + Env(gp41)-E2 immunized rabbits developed binding Abs specific to the N-terminal MPER peptide overlapping the 2F5 epitope. Minimal Ab responses were detected to the C-terminal MPER peptide overlapping the 4E10 and 10E8 epitopes. One plausible explanation for decreased binding Abs specific for the C-terminal MPER peptide compared to the N-terminal MPER peptide in rabbits immunized with Env(MPER)-E2 particle is the proximity of the C-terminal peptide to the E2 scaffold itself. The N-terminal MPER epitope is closer to the N-terminus of the recombinant particles, whereas the C-terminal epitope was in closer proximity to the E2 scaffold, and steric hindrance of the scaffold may have played a role in the diminished response to the C-terminal epitope. SPR analysis demonstrated binding of the 2F5 mAb to the Env(MPER)-E2 particles, but a lack of binding of 4E10 and 10E8 mAbs, providing evidence of inaccessibility of these epitopes on the surface of the scaffold. Adding residues between these mAb epitopes and the E2 scaffold may increase the access to this region, which may in turn result in better responses. In theory, it should be possible to further optimize immune responses to conserved epitopes by modifying the presentation of the MPER peptide on the E2 scaffold by either altering the residues or grafting the peptide onto the scaffold in a constrained conformation, similar to studies displaying V3 on the cholera toxin B scaffold [63] and chimeric rhinovirus displaying MPER [35], [36]. Another potential reason for minimal C-terminal MPER-specific Abs may be the absence of the transmembrane (TM) domain of gp41. Recent studies have suggested that the gp41 TM domain plays a pivotal role in orienting the 4E10 epitope [64], and therefore molecular modeling of this region may be necessary to optimize presentation on the E2 scaffold.

NAbs were detected in all rabbits immunized with Env gp160 DNA plus Env(MPER)-E2 particles; however, the majority of the NAb response in rabbits immunized with gp160 bearing V3 was directed to the immunodominant V3 region. V3 has structural conservation and can serve as a target for inter-clade NAbs [65]; however, this epitope has also been shown to have considerable sequence diversity among clades of HIV-1 [66]. It has been suggested that V3 may serve as a decoy to distract the immune system from more conserved regions of Env such as the MPER, and contribute to the production of ineffective humoral responses that are clade- or strain-specific [11]. We found that we could overcome the immunodominance of V3 by removing it from the gp160 construct (Env-gp160ΔV3) and thereby broaden Ab recognition of other epitopes. Significantly increased autologous NAbs were detected after 28 weeks however, no other major improvements to NAb breadth or neutralization of the chimeric viruses were measured.

Ab responses directed to the V2 region have been shown to be an important correlate in the RV144 Thai trial [67], significantly raising interest in V2 as an immunogen. However, removal of the V1V2 region or of the V2 loop alone from Env of HIV-1 or SIV increases the sensitivity of these viruses to NAbs, and removal of the V2 loop enhanced the neutralization susceptibility of SF162 to the NmAbs 2F5 and 4E10 [44]. Studies in macaques revealed that a ΔV2gp140 immunogen elicited the highest gp41 Ab titers of all the gp140 immunogens tested [68], although NAb responses were not improved [45]. These prior studies prompted us to construct the gp160ΔV2 DNA immunogen in order to determine if co-immunization with Env(MPER)-E2 particles could elicit NAbs redirected to MPER. We speculated that these immunogens might also elicit responses that further target V3, and therefore we also constructed gp160ΔV123 DNA to eliminate both V2 and V3 responses.

Using these new constructs of gp160 full-length or variable loop deleted DNA in combination with Env(MPER)-E2 particles, we found that twelve out of sixteen rabbits developed MPER-directed NAbs as measured by HIV-2/HIV-1 MPER chimeric viruses. These values were similar to the IC_50_ values against HIV-SF162. Although these concentrations do not reach the same potency level as the NmAbs 2F5 or 4E10, these concentrations for purified polyclonal IgG are significantly less than doses of passively transferred polyclonal IgG required for protection in macaque studies (typically 100–200 mg/kg or 1–2 mg/ml) [69]. HIV-2/MPER chimera neutralization could not be competed to any significant degree with the MPER peptides that we tested. Thus we could not definitively prove that the NAbs are directed to the specific MPER found in these HIV-2 chimeras. Although rabbits within the gp160ΔV3 group developed significantly higher binding Abs specific to MPER and high SF162 NAbs, sera from these rabbits did not have significantly higher NAbs against the HIV-2/HIV-1 MPER chimeras. We have not tested the Env(MPER)-E2 particles alone to determine if they can elicit these MPER specific responses without co-immunizing with gp160 DNA due to previous studies where we showed that Env(V3)-E2 particles were very poor at generating NAbs when used alone [39].

Interestingly, the MPER-specific binding Abs elicited by the various regimens required the SLWN residues at position 667–70 to detect MPER-specific binding for the gp160wt and gp160ΔV2 groups. Abs from the gp160ΔV3 and gp160ΔV123 groups bound to slightly different specificities, where weak binding was observed to the DKWA epitope. A prior study by Menendez, et al. found that 2F5 binding to MPER peptides was not dependent on the serine at position 667, but instead binding strength increased as a result of a hydrophobic residue three residues C-terminal to the tryptophan in DKW, in which leucine or tryptophan residues were preferentially selected [70]. If the MPER-specific neutralization epitope also requires the SLWN residues, then this epitope does not overlap with the NmAbs, 2F5, 4E10 or 10E8, suggesting that targeting of the MPER epitope was varied compared to these bNmAbs. These data suggest that the broadly reactive neutralization determinants are either closer to the membrane or conformational, and our current Env(MPER)-E2 particles limit accessibility of these epitopes, as supported by the SPR analysis.

Because we had some evidence for MPER-specific targeting using these particles, we wanted to determine the breadth of the neutralization. We measured modest neutralization of Tier 1 and 2 viruses in all immunization groups, with most rabbit serum neutralizing 2–3 isolates in addition to SF162. These NAb responses varied following each immunization, with the highest breadth observed early in the vaccination schedule and decreasing over time for the gp160wt, gp160ΔV3 and gp160ΔV2 groups, similar to binding Abs recognizing MPER (Fig. 5A). Notably, low-level neutralization against JRCSF was detected in five rabbits at 6 and 14 weeks, while no neutralization against JRCSF was detected at 28 weeks. The gp160ΔV123 group had increased potency of SF162 and cross-neutralization breadth after four immunizations, providing evidence that combining this construct with optimized Env(MPER)-E2 particles may result in enhanced neutralization breadth and potency.

Directing responses to the conserved MPER region has been difficult, despite numerous efforts [31]–[33], [34]–[36]. In the experiments described here, we have evidence that Abs are being directed to the MPER. However, although MPER binding is strong, and neutralization of multiple strains is improved by the MPER-E2 immunogen, the contribution of MPER reactivity to neutralization cannot be determined. Our data indicate that the Env(MPER)-E2 particles are capable of, eliciting low-level cross-NAb responses when co-immunized with unmodified gp160 DNA or deletions of hypervariable regions V1–V3, all of which express the MPER region in the DNA. The strength of this conclusion is based upon the relatively large number of animals tested and responding (25 total), the rigorous assays using purified IgG from individual rabbits to show neutralization specificity, and the modest breadth of neutralization against both Tier 1 and Tier 2 viruses. The concentration of HIV-SF162 NAbs did not predict which sera would have breadth, but on the whole IC_50_ values dropped following 3, 4 and 5 immunizations showing increased potency of the responses. We consider it promising that the three of the four rabbits with neutralizing activity against more than one of the HIV-2 chimeras (M7100, M7107, and M7109) also had NAbs against the 4–6 of the panel of 7 heterologous viruses. The basis of this neutralization may well differ in each of the vaccine groups. We are encouraged by these results when compared to other gp41-based scaffolds, as the potency of the heterologous NAbs (IC_50_ values ranging from the low to mid 100 s) is similar to those found in other published studies [71] and the breadth is greater. Clearly our MPER-based immunogen requires further optimization for advancement. We hypothesize that the responses can be further enhanced with modifications of the MPER presentation on the E2 particles, as well as changes to the vaccine regimen, such as boosting with the MPER particles alone. Once MPER-specific responses are primed by the particle plus DNA immunizations, the Env(MPER)-E2 particles might be very effective as boosting agents, a strategy that we propose for future studies. Utilization of this multimeric scaffold is a promising strategy to target known neutralization determinants, and provides a basis for further development of a broadly reactive HIV-1 vaccine.

## Supporting Information

S1 Figure **Expression of gp160 Env variants.** (A) Western blot analysis of Env variants expressed in 293T cells post-transfection. Total cell lysate was loaded onto the gel and subsequently probed using CHIVIG polyclonal antibodies. (B) Cell surface expression of the Env variants after transfection into COS-7 cells. Immunofluorescence was detected using CHIVIG and FITC-conjugated goat anti-human IgG. The presence of the expressed Env variants was visualized on the cell surface using fluorescence microscopy.
(DOCX)

S2 Figure **Longitudinal linear antibody epitope mapping.** Binding of antibodies directed to linear epitopes of Env elicited in rabbits co-immunized with SF162 gp160wt, gp160ΔV3, gp160ΔV2, gp160ΔV123 DNA and Env(MPER)-E2 was determined by linear peptide ELISA. The clade B consensus Env peptides (15-mer with 11 aa overlap) were used to assess responses from pooled sera at weeks 6, 12, 22, and 28 at a dilution of 1∶25. Responses were considered positive if they achieved OD values two-fold over pre-immune sera values. Colors indicate responses of sera from week 6 (gray, 2 weeks post second immunization), week 14 (red, 2 weeks post third immunization), week 22 (green, 2 weeks post fourth immunization), and week 28 (blue, 2 weeks post fifth immunization).
(DOCX)

S3 Figure **Serological responses following three immunizations.** Binding of antibodies directed to MPER peptides in rabbits co-immunized with SF162 gp160wt, gp160ΔV3, gp160ΔV2, gp160ΔV123 DNA and Env(MPER)-E2 was determined by linear peptide ELISA. Neutralization (IC_50_) against the HIV-2/HIV-1 MPER chimeras C1, C2, and C3 and six HIV-1 pseudoviruses is displayed for individual rabbit serum samples. Colors indicate the potency of the responses as denoted in the key to the right. HIV-1 insert sequences of the C1, C2, and C3 viruses are shown below the table.
(DOCX)
