# Correction: Multimeric Scaffolds Displaying the HIV-1 Envelope MPER Induce MPER-Specific Antibodies and Cross-Neutralizing Antibodies when Co-Immunized with gp160 DNA

**DOI:** 10.1371/journal.pone.0120027

**Published:** 2015-03-27

**Authors:** The PLOS ONE Staff

The eleventh author’s name is spelled incorrectly. The correct name is: Piergiuseppe De Berardinis.
