# Supplementary figures and images for: Multimeric Scaffolds Displaying the HIV-1 Envelope MPER Induce MPER-Specific Antibodies and Cross-Neutralizing Antibodies when Co-Immunized with gp160 DNA
Source: PLoS One. 2014 Dec 16;9(12):e113463. doi: 10.1371/journal.pone.0113463 (PMC4267727; doi:10.1371/journal.pone.0113463)

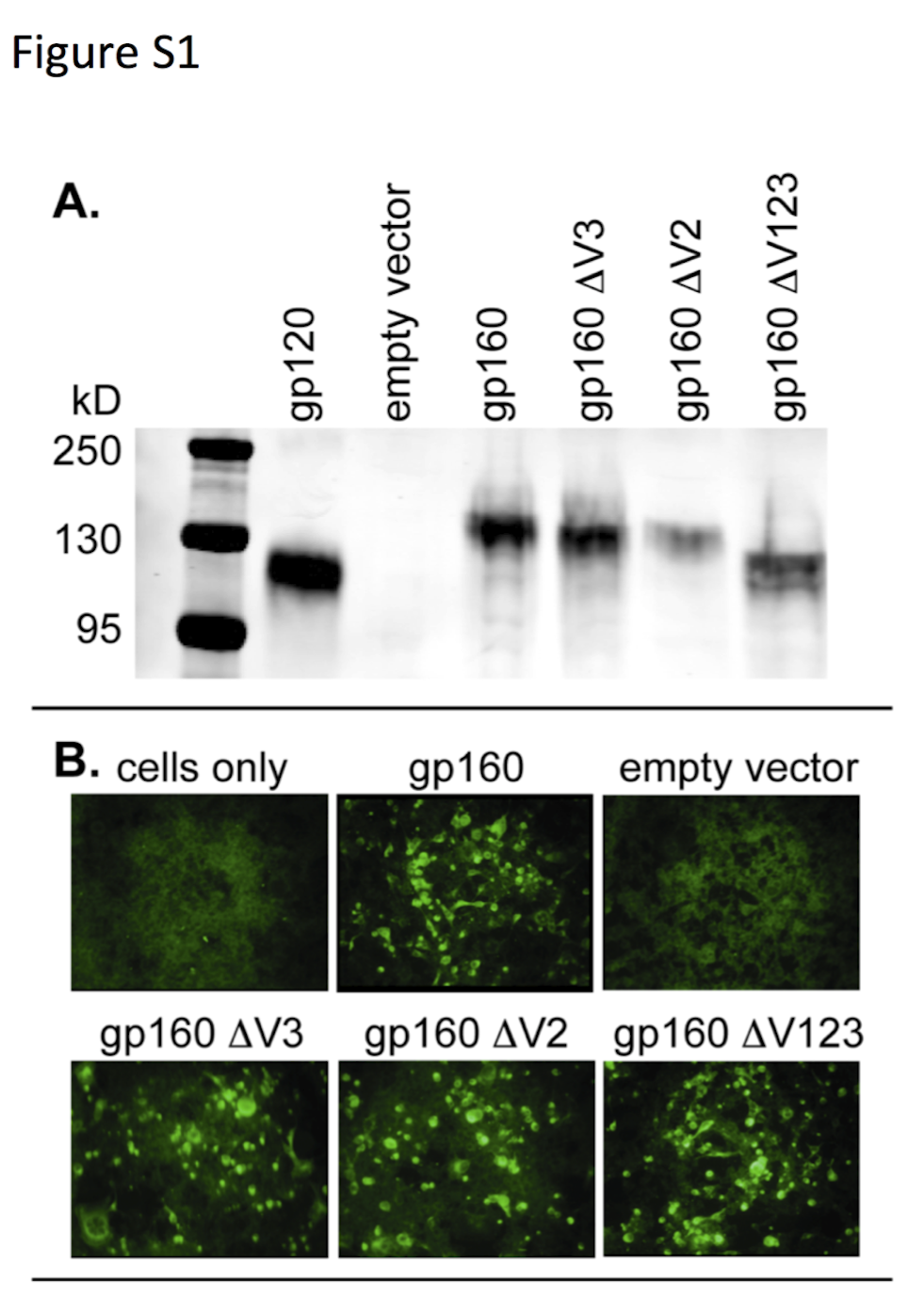

Supplement: S1 Figure — Expression of gp160 Env variants. (A) Western blot analysis of Env variants expressed in 293T cells post-transfection. Total cell lysate was loaded onto the gel and subsequently probed using CHIVIG polyclonal antibodies. (B) Cell surface expression of the Env variants after transfection into COS-7 cells. Immunofluorescence was detected using CHIVIG and FITC-conjugated goat anti-human IgG. The presence of the expressed Env variants was visualized on the cell surface using fluorescence microscopy. (DOCX) [file pone.0113463.s001.docx]

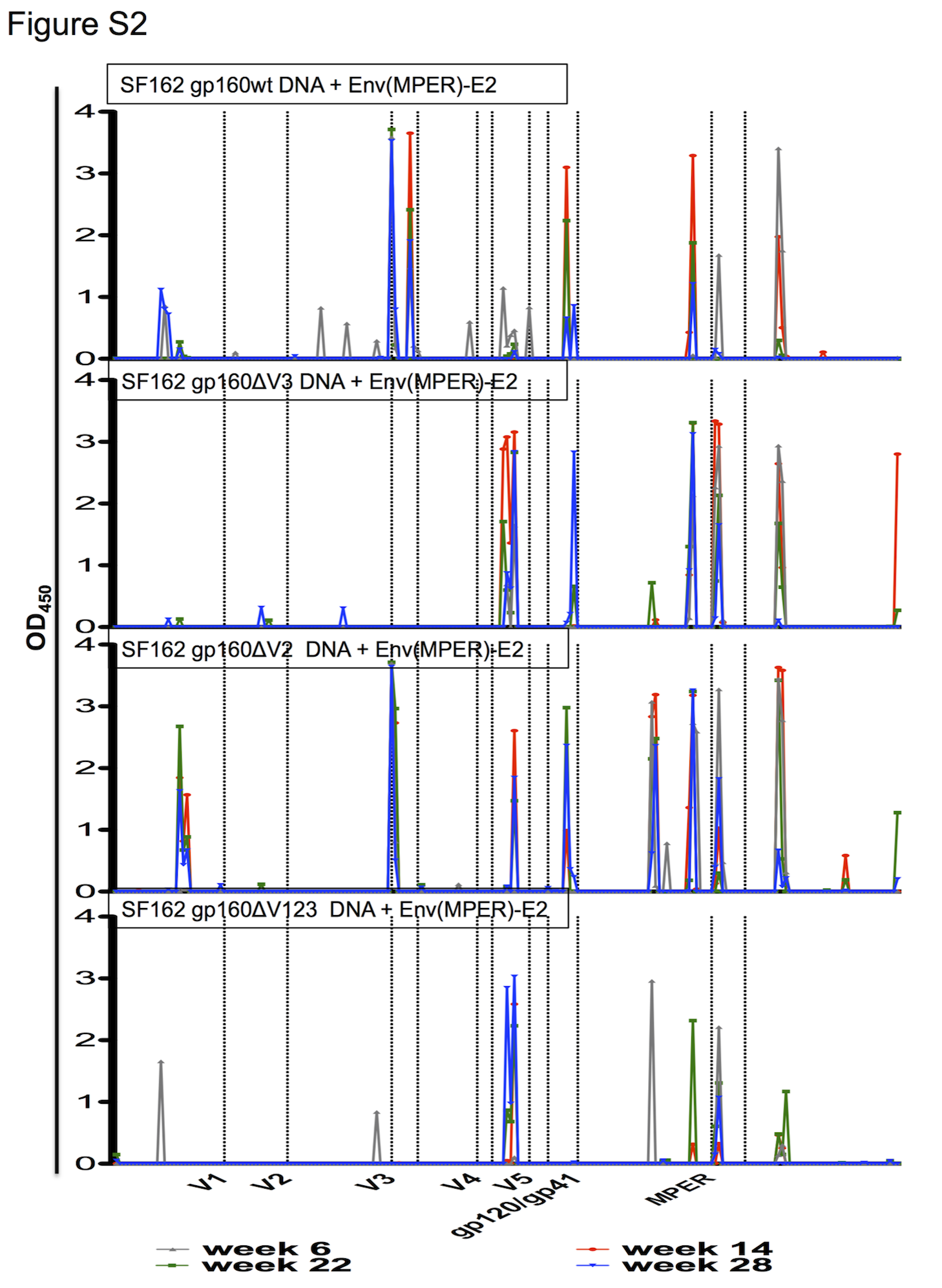

Supplement: S2 Figure — Longitudinal linear antibody epitope mapping. Binding of antibodies directed to linear epitopes of Env elicited in rabbits co-immunized with SF162 gp160wt, gp160ΔV3, gp160ΔV2, gp160ΔV123 DNA and Env(MPER)-E2 was determined by linear peptide ELISA. The clade B consensus Env peptides (15-mer with 11 aa overlap) were used to assess responses from pooled sera at weeks 6, 12, 22, and 28 at a dilution of 1∶25. Responses were considered positive if they achieved OD values two-fold over pre-immune sera values. Colors indicate responses of sera from week 6 (gray, 2 weeks post second immunization), week 14 (red, 2 weeks post third immunization), week 22 (green, 2 weeks post fourth immunization), and week 28 (blue, 2 weeks post fifth immunization). (DOCX) [file pone.0113463.s002.docx]

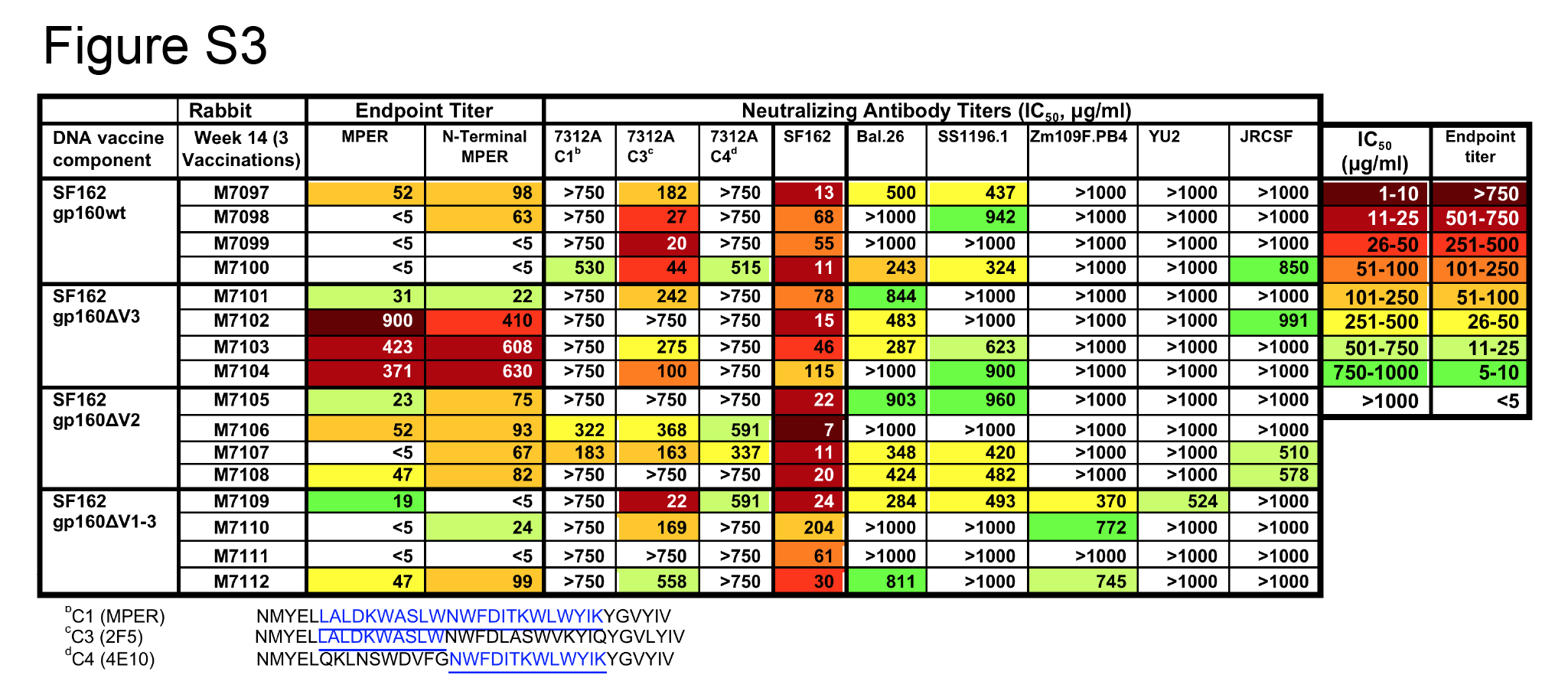

Supplement: S3 Figure — Serological responses following three immunizations. Binding of antibodies directed to MPER peptides in rabbits co-immunized with SF162 gp160wt, gp160ΔV3, gp160ΔV2, gp160ΔV123 DNA and Env(MPER)-E2 was determined by linear peptide ELISA. Neutralization (IC50) against the HIV-2/HIV-1 MPER chimeras C1, C2, and C3 and six HIV-1 pseudoviruses is displayed for individual rabbit serum samples. Colors indicate the potency of the responses as denoted in the key to the right. HIV-1 insert sequences of the C1, C2, and C3 viruses are shown below the table. (DOCX) [file pone.0113463.s003.docx]
